# hapCon: estimating contamination of ancient genomes by copying from reference haplotypes

**DOI:** 10.1093/bioinformatics/btac390

**Published:** 2022-06-13

**Authors:** Yilei Huang, Harald Ringbauer

**Affiliations:** Department of Archaeogenetics, Max Planck Institute for Evolutionary Anthropology, 04103 Leipzig, Germany; Department of Archaeogenetics, Max Planck Institute for Evolutionary Anthropology, 04103 Leipzig, Germany

## Abstract

**Motivation:**

Human ancient DNA (aDNA) studies have surged in recent years, revolutionizing the study of the human past. Typically, aDNA is preserved poorly, making such data prone to contamination from other human DNA. Therefore, it is important to rule out substantial contamination before proceeding to downstream analysis. As most aDNA samples can only be sequenced to low coverages (<1× average depth), computational methods that can robustly estimate contamination in the low coverage regime are needed. However, the ultra low-coverage regime (0.1× and below) remains a challenging task for existing approaches.

**Results:**

We present a new method to estimate contamination in aDNA for male modern humans. It utilizes a Li&Stephens haplotype copying model for haploid X chromosomes, with mismatches modeled as errors or contamination. We assessed this new approach, hapCon, on simulated and down-sampled empirical aDNA data. Our experiments demonstrate that hapCon outperforms a commonly used tool for estimating male X contamination (ANGSD), with substantially lower variance and narrower confidence intervals, especially in the low coverage regime. We found that hapCon provides useful contamination estimates for coverages as low as 0.1× for SNP capture data (1240k) and 0.02× for whole genome sequencing data, substantially extending the coverage limit of previous male X chromosome-based contamination estimation methods. Our experiments demonstrate that hapCon has little bias for contamination up to 25–30% as long as the contaminating source is specified within continental genetic variation, and that its application range extends to human aDNA as old as ∼45 000 and various global ancestries.

**Availability and implementation:**

We make hapCon available as part of a python package (hapROH), which is available at the Python Package Index (https://pypi.org/project/hapROH) and can be installed via pip. The documentation provides example use cases as blueprints for custom applications (https://haproh.readthedocs.io/en/latest/hapCon.html). The program can analyze either BAM files or pileup files produced with samtools. An implementation of our software (hapCon) using Python and C is deposited at https://github.com/hyl317/hapROH.

**Supplementary information:**

Supplementary data are available at *Bioinformatics* online.

## 1 Introduction

In recent years, ancient DNA (aDNA) has become a new powerful scientific instrument for studying the human past. However, aDNA is often highly fragmented and degraded, and the amount of endogenous DNA is typically low. Therefore aDNA is particularly prone to contamination from other human DNA, in particular during excavating and handling samples and extracting aDNA. Ruling out substantial contamination before proceeding to downstream analysis is a critical quality control step. This task requires reliable tools to estimate contamination rates for low coverage aDNA.

One widely used approach to estimate contamination for aDNA utilizes heterozygosity in mitochondrial genomes (mtDNA) as an individual’s mtDNA is haploid; therefore, apparent heterozygous sites on mtDNA contain evidence about contamination (Renaud *et al.*, 2015, e.g. Schmutzi). For most ancient samples, mtDNA can be sequenced to relatively high coverage, facilitating such analysis. However, the ratio of preserved endogenous mtDNA to nuclear DNA varies substantially across samples, creating a complex relationship between mtDNA and nuclear DNA contamination. A sample can be highly contaminated for its nuclear DNA but minimally contaminated for its mtDNA, and vice versa (Furtwängler *et al.*, 2018).

Various general approaches for estimating nuclear contamination exist. ContamLD, for example, utilizes breakdown of linkage disequilibrium introduced by contaminant sequences to estimate contamination rate since the contaminant haplotype is uncorrelated with the endogenous haplotype (Nakatsuka *et al.*, 2020). It requires comparably high coverage [∼0.5× for 1240k data and ∼0.1× for whole genome sequencing (WGS) data], which is not readily available for many of the aDNA samples sequenced so far. Another recently introduced method, AuthentiCT, uses post-mortem damage pattern to estimate contamination (Peyrégne and Peter, 2020). It can work with very low coverage samples, but is designed for a specific laboratory protocol (single-stranded DNA libraries without UDG treatment), limiting its usage to only a small fraction of aDNA data. Moreover, AuthentiCT cannot detect contamination originating from ancient sources. The software DICE performs joint estimate of demography, sequencing error and contamination rate but it requires very high coverage (∼3×), which is not obtained for the vast majority of aDNA samples (Racimo *et al.*, 2016).

For male samples, an approach to estimate nuclear contamination well suited for low-coverage data exists. It exploits the naturally haploid male X chromosome, similar to estimating contamination in mtDNA. Several methods have been developed to utilize this signal (Moreno-Mayar *et al.*, 2020, e.g. Rasmussen *et al.*, 2011). All of them require sites covered by at least two aligned sequences to measure heterozygosity. However, for low-coverage data most covered sites are covered by one aligned sequence only. Assuming that sequencing depth at each site follows Poisson distribution per site, for 0.1× average genome-wide coverage about 0.47% sites is expected to be covered by at least two sequences, for 0.05× dropping to 0.12% and for 0.01× to only 0.005%. As a result, only a small fraction of sequence data can be used for estimating contamination, causing the estimates for ultra-low coverage samples to be highly variable with wide confidence intervals.

Here, we present a new approach to estimate contamination rates based on haplotype copying on male X chromosome that also utilizes sites covered by only one sequence. We model the X chromosome of the endogenous individual as a mosaic copy from a modern reference panel, and model sporadic mismatches of observed sequences from the copied haplotypes as either errors or contamination. An implementation of the new method is available as Python package (hapCon, https://pypi.org/project/hapROH). Using a Hidden Markov Model (HMM), the software estimates contamination by maximum likelihood. Extensive simulation and down-sampling experiments demonstrate that hapCon produces estimates with smaller variance and narrower confidence intervals than previous methods using the male X chromosome. It substantially extends the application range of contamination estimates on male samples, yielding reliable results for as low as 0.1× coverage on a widely used aDNA data type (1240k capture) and for as low as 0.02× WGS data (all coverages refer to average sequencing depth on the X chromosome).

## 2 Materials and methods

The core of our new method is a HMM haplotype copying approach widely used in genomics (Li and Stephens, 2003, Li&Stephens) that models a haplotype as a mosaic of haplotypes from a reference panel. Since X chromosomes of males are haploid, they can be naturally modeled as such a haplotype mosaic without phasing, which would be particularly challenging for low-coverage data. Any aligned sequences discordant from the copied haplotype can be due to various causes (including mutation, gene conversion, sequencing error, aDNA post-mortem damage or contamination), but only contamination mismatches correlate with population allele frequencies. We utilize this signal within the Li&Stephens HMM by incorporating the single locus contamination model of ANGSD (Rasmussen *et al.*, 2011).

### 2.1 The Hidden Markov Model

**Fig. 1.**
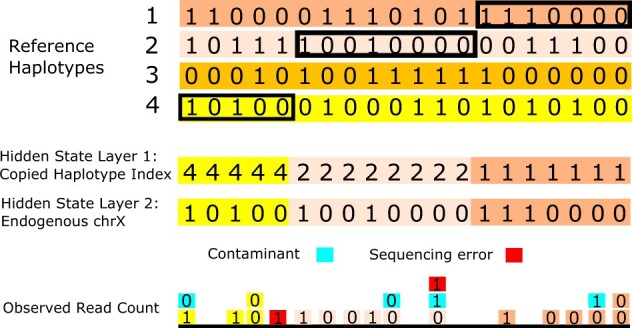
Graphical illustration of the model to estimate contamination rates via copying haplotypes from a haplotype reference panel. The target male X chromosome is modeled as a mosaic copy from a haplotype reference panel. In this specific case, the haplotype is copied from reference haplotype 4,2,1 (from left to right). The observed read counts at each biallelic marker are modeled as a mix of sequences from the endogenous and contaminant haplotypes and sequencing errors

Throughout, we model biallelic markers on haploid X chromosomes. For each marker, the Li&Stephens HMM has *n* hidden states given *n* haplotypes from a reference haplotype panel. A marker being in state i(1≤i≤n) denotes its genotype being copied from the reference haplotype indexed by *i* (Fig. 1). This general Li&Stephens HMM is then fully specified by setting transition probabilities between markers and emission probabilities for the genotype data. Here, we use a standard transition model with jump probabilities depending on the genetic map distance between markers as measured in Morgan. For the emission probabilities of aligned sequences supporting the reference and alternative alleles, we adapt the previously published ANGSD model (Rasmussen *et al.*, 2011).

### 2.2 Transition probabilities

We define the transition probability between hidden states for each pair of adjacent markers l,l+1 as in Ringbauer *et al.* (2021). Given an infinitesimal rate matrix *Q* of dimension *n *×* n*, the full transition probability matrix between marker *l* and *l *+* *1 is obtained by exponentiation of the rate matrix: $T_{l\to l+1}=\text{exp}(Q\cdot r_{l})$, where *r_l_* denotes the genetic map distance between marker *l* and *l *+* *1 (measured in Morgan). We assume that each reference haplotype has an equal prior probability to be copied from, therefore a single rate *q* fully specifies off-diagonal elements of *Q*, and $Q_{ii}=-(n-1)q$. We set *q *=* *300, see Supplementary Note S2.3.3 for further details.

### 2.3 Emission probabilities

Assume we have known genotype data $i_{1},\ldots ,i_{L}$ at *L* biallelic markers along the *i*th haplotype in the reference panel, with two possible values 0 and 1 encoding reference and alternative alleles, respectively. At each marker *l*, we model the so-called read counts, defined as the number of mapped sequences aligned to that genomic position that support either the reference or the alternative allele. Throughout, we use the term ‘read counts’ in a broad sense to refer to the number of aligned DNA sequences that potentially have undergone pre-processing steps such as adaptor trimming, paired-end read merging and PCR deduplication.

To model mismatches between the observed genotype data and the copied haplotype, we introduce three mismatch parameters: $\epsilon_{g},\epsilon_{r}$ and *c*. First, *ϵ_g_* is the error rate per base, denoting the probability of a single base being erroneously altered. This rate can be estimated from monomorphic sites adjacent to polymorphic sites. This term models several sources of errors, including sequencing error, aDNA characteristic sequence damage and mismapping (see Supplementary Notes S2.3.1 and S2.3.4 for details). Second, the so-called mis-copying error rate *ϵ_r_* is an aggregate error term to model mismatches between the endogenous haplotype and the copied haplotype due to various causes (including mutation, gene conversion, errors in the reference panel, etc.). This mis-copying error model is widely used in phasing and imputation algorithms based on the Li&Stephens model (Browning *et al.*, 2021, e.g. Delaneau *et al.*, 2019; Loh *et al.*, 2016; Rubinacci *et al.*, 2021). We fix $\epsilon_{r}=1e^{-3}$, as preliminary tests indicated that this value provides good performance on simulated and empirical aDNA data while also providing some flexibility so that the copying path is not truncated by errors (see Supplementary Note S2.3.2 for details). Third, the contamination rate *c* models the fraction of the sequences originating from contamination, which is the parameter we wish to estimate.

We use a two-layer approach to model the observed read counts of the endogenous haplotype at each marker. The first layer models the endogenous haplotype given the copying state, and the second layer describes how sequences are drawn given the endogenous haplotype.

The first layer specifies the genotype probability $t_{l}\in \{0,1\}$ of the endogenous haplotype for each marker *l*, given the underlying copying state, $s_{l}\in \{1,2,\ldots ,n\}$. Haplotype copying with copying error rate *ϵ_r_* gives:

(1) $$p(t_{l}=0|s_{l}=i)=(1-\epsilon_{r})1_{i_{l}=0}+\epsilon_{r}1_{i_{l}=1},$$

where $1_{i_{l}=0}$ is the indicator variable that takes value 1 when the reference haplotype *i* carries allele 0 at marker *l* and 0 otherwise ($1_{i_{l}=1}$ is defined analogously). The probability for the alternative allele $p(t_{l}=1|s_{l}=i)$ is obtained similarly.

The second layer then models the probability of a single sequence supporting the alternative allele given the latent genotype. Let *c* denote the genome-wide contamination rate, *p_l_* the alternative allele frequency in the contaminating population at marker *l*. Then the probability of a sequence supporting the alternative allele is

(2) $$\begin{array}{c} p(\text{alternative}|t_{l}=0)=(1-c)\epsilon_{g}+c\left(p_{l}(1-\epsilon_{g})+(1-p_{l})\epsilon_{g}\right),\\ p(\text{alternative}|t_{l}=1)=(1-c)(1-\epsilon_{g})+c\left(p_{l}(1-\epsilon_{g})+(1-p_{l})\epsilon_{g}\right). \end{array}$$

The probability for a single sequence base being alternative given the hidden state *s_l_* is obtained by combining the two layers and summing over the two possible latent genotypes:

(3) $$\begin{array}{c} p(\text{alternative}|s_{l})=p(\text{alternative}|t_{l}=0)p(t_{l}=0|s_{l})\\ +\;p(\text{alternative}|t_{l}=1)p(t_{l}=1|s_{l}). \end{array}$$

Finally, we model the read counts by a binomial distribution fully determined by the probability of a single sequence base being alternative. Let $c_{l_{0}},c_{l_{1}}$ denote the number of aligned sequences supporting reference and alternative alleles, respectively. Denoting the total sequencing depth at marker *l* as $n=c_{l_{0}}+c_{l_{1}}$ and abbreviating $p_{\mathrm{alt}}(s_{l})=p(\text{alternative}|s_{l})$ gives:

(4) $$p(c_{l_{0}},c_{l_{1}}|s_{l})=\left(\begin{array}{c} n\\ c_{l_{1}} \end{array}\right){\left(1-p_{\text{alt}}(s_{l})\right)}^{n-c_{l_{1}}}p_{\text{alt}}{\left(s_{l}\right)}^{c_{l_{1}}}.$$

This probability of the observed read counts for each HMM state fully specifies the emission probabilities of the HMM.

### 2.4 Maximum likelihood estimation

For a given contamination rate *c* and with all other parameters set, we then use a standard scaled forward algorithm to calculate the overall likelihood of the HMM model (Bishop, 2006). To obtain a maximum likelihood estimate $\hat{c}$ of *c*, we use the iterative method L-BFGS-B (Byrd *et al.*, 1995; Zhu *et al.*, 1997) provided in SciPy (Virtanen *et al.*, 2020) searching within the interval [0,0.5]. We estimate the standard error of the MLE estimate $\hat{c}$ by numerically calculating Fisher Information of the likelihood function around $\hat{c}$ using the Python package numdifftools, and then approximate the 95% confidence interval by ±1.96× standard errors. Since our model is not defined for *c *<* *0, for $\hat{c}=0$ the first derivative may not be zero at $\hat{c}=0$ and thus confidence intervals cannot be approximated with the Fisher Information matrix alone. Instead, we use quadratic interpolation based on first and second derivatives with *c* to approximate the likelihood function around $\hat{c}=0$ and use the set of parameters whose likelihood is at least 14.7% of the maximum likelihood to obtain 95% confidence intervals (the so-called ‘14.7% likelihood region’, see Supplementary Note S1 for details)

### 2.5 Implementation and runtime

We prepared two reference panels tailored toward two common aDNA data types. The first panel contains all sites in the widely used enrichment capture strategy consisting of ca. 1.2 million SNPs, henceforth referred to as ‘1240k’ panel (Fu *et al.*, 2015; Haak *et al.*, 2015; Mathieson *et al.*, 2015). The second panel contains all biallelic sites in the WGS 1000Genome dataset (Auton *et al.*, 2015) with minor allele frequency (MAF) greater than 5%, henceforth referred to as ‘1000G’ panel. We chose this 5% MAF filter because initial exploratory analysis indicated that this cutoff provides a robust trade-off between accuracy and run time (Supplementary Fig. S25). We explored MAF ranging from 0.2% to 20% and found that the width of confidence interval increases only slightly when increasing MAF cutoff, suggesting that most signal comes from common variants.

We implemented hapCon as a Python package, expanding upon code from the software hapROH which uses a similar copying HMM (Ringbauer *et al.*, 2021). We verified the correctness of our implementation by performing under the model simulation (Supplementary Notes S2.1, S2.2 and Supplementary Figs S1 and S2). We measured our method’s runtime [including preprocessing time to parse BAM file with samtools (Li *et al.*, 2009; Li, 2011)] on Intel(R) Xeon(R) Gold 6240 CPU @ 2.60 GHz on WGS data with coverages ranging from 0.02× to 5× (Supplementary Fig. S38). As expected, the run time grows approximately linearly with the number of sites covered by at least one sequence. The run time of our method with 1000G panel remains within three minutes for a typical aDNA sample with coverage less than 1×, making our new method viable for any large-scale aDNA studies. Our benchmarking experiment also shows that our method is four times slower with 1000G panel than with 1240k panel, as expected since the 1000G panel contains about four times more SNPs than the 1240k panel. For comparison, we used the C++ version of ANGSD. The results indicate that our method is faster than ANGSD at coverage higher than 1×.

### 2.6 Relations to previous methods

Several methods that utilize heterozygous sequences in haploid regions to estimate contamination rates in aDNA data have been developed. ANGSD, a widely used method, assumes that true endogenous allele is supported by the majority of the aligned sequences at a site (Rasmussen *et al.*, 2011). More recently, a similar approach has been developed which assigns equal priors to both the reference and alternative alleles (Moreno-Mayar *et al.*, 2020, two-consensus method). Our new approach can be considered as a many-consensus model where the true endogenous allele originates from a set of reference haplotypes and each of them being weighted by the Li&Stephens haplotype copying framework that utilizes linkage information. We note that in the limit of widely spaced markers and consequently little linkage information, our model converges to the two-consensus approach, but with priors according to the allele frequency in the reference panel.

We hypothesized that our method’s performance gain is driven by its ability to utilize sites covered by only one sequence. Such data can be used by neither ANGSD nor the two-consensus approach as both need at least two sequences per site to establish evidence of contamination. In contrast, our method can detect potential contamination via comparing single sequence to the copied reference haplotypes. As a proof of concept, we simulated read counts and down-sampled every covered site to exactly one sequence (see Supplementary Note S2.2 for details). Our results demonstrate that our method can still produce accurate contamination estimates, even when relying only on this so-called pseudohaploid data (Supplementary Fig. S2).

## 3 Results

We assessed the performance of our new approach, hapCon, on both simulated and empirical aDNA data. Throughout our tests, we set the following default settings. We used a reference panel consisting of all non-African haplotypes from the 1000Genome Project (Auton *et al.*, 2015) (see Section 3.2.2 for the detailed rationale) except when analyzing endogenous sources with known African ancestry where we use the full 1000Genome reference panel. Unless specified otherwise, we set allele frequencies of the CEU individuals (CEU: Northern Europeans from Utah in 1000 Genome panel) as the proxy for the contamination source allele frequency. For comparison, we used ANGSD’s Method 1 (new_llh) with default settings. We filtered aligned aDNA sequences to mapping quality greater than 30 and to base quality greater than 20. For each simulated scenario, we generated 100 independent replicates. For every replicate, we report the maximum likelihood point estimate of the contamination rate and a 95% confidence interval.

### 3.1 Assessing performance on simulated contaminated data

#### 3.1.1 Testing on a range of ancestries and contamination levels

**Fig. 2.**
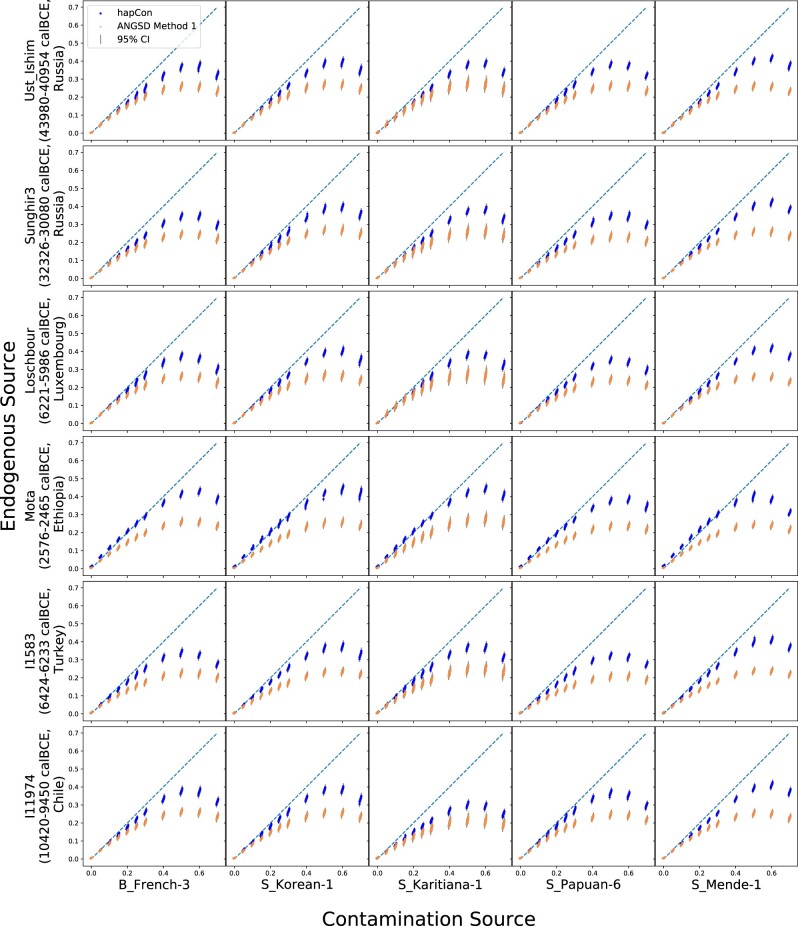
Performance of hapCon on simulated contamination using various endogenous and contamination sources. We compared hapCon and ANGSD at 0.5× coverage with various endogenous and contamination sources. For each simulated scenario, we generated 100 independent replicates and visualized the point estimates and confidence intervals obtained from hapCon with 1240k panel and ANGSD. For each of the contaminant source B_French-3, S_Korean, S_Karitiana-1, S_Papuan-6 and S_Mende-1, we used allele frequencies of CEU (Utah residents with Northern and Western European ancestry), CHB (Han Chinese in Beijing, China), MXL (Mexican Ancestry in Los Angeles CA USA), CHB, YRI (Yoruba in Ibadan, Nigeria) from the 1000Genome Project as the proxy for the allele frequency of the contaminant population. Better proxies for S_Karitiana-1 and S_Papuan-6 exist, for example, PEL (Peruvian in Lima, Peru) and KHV (Kinh in Ho Chi Minh City, Vietnam). However, the reference panels provided by ANGSD are prepared from HapMap (Consortium *et al.*, 2003), which is only a subset of 1000Genome. To ensure a fair comparison, we used CHB and MXL for S_Papaun-6 and S_Karitiana-1 for hapCon as well. A zoom-in into the simulated contamination in the range of 0–10% is available at Supplementary Figure S22

We first assessed our new method on simulated samples with artificial contamination created by mixing two BAM files from different individuals. To investigate how the ancestry of the endogenous and the contaminant haplotypes affects contamination estimates, we used as the endogenous source Ust Ishim [43980-40954 calBCE, Russia (Fu *et al.*, 2014)], Sunghir3 [33685-31328 calBCE, Russia (Sikora *et al.*, 2017)], Loschbour [6221-5986 calBCE, Luxembourg (Lazaridis *et al.*, 2014)], Mota [2576-2465 calBCE, Ethiopia (Llorente *et al.*, 2015)], I1583 [6424-6233 calBCE, Turkey (Mathieson *et al.*, 2015)] and I11974 [10420-9450 calBCE, Chile (Posth *et al.*, 2018)], and as the contaminant source B_French-3, S_Korean, S_Karitiana-1, S_Papuan-6 and S_Mende-1 from the Simons Genome Diversity Project (Mallick *et al.*, 2016), creating 30 different combinations. This experiment is designed to test a wide variety of endogenous sources, including one of the oldest modern humans sequenced so far (Ust Ishim), under-represented ancestries such as African and Native American ancestries, and samples representative of the majority of aDNA data (hunter-gatherers, Neolithic farmers and Steppe pastoralists), while the contaminant sources include representatives from all continental populations. To determine how much contamination our haplotype copying model can tolerate, we simulated contamination ranging from 0% to 70% (with steps of 5% within range 0–30%, and with steps of 10% for range 40–70%). We simulated 100 independent replicates at 0.5× average coverage for each scenario and summarized results in Figure 2.

When comparing our method with ANGSD, we found that both methods underestimate contamination rate for highly contaminated samples (i.e. >20% contamination); however, hapCon estimates are less biased (Fig. 2). We note that this bias for substantially contaminated samples is often tolerable in practice because, as long as a sample is highly contaminated, the exact rate of contamination is not of general interest since such samples are usually excluded from downstream analysis or at least filtered to only sequences with aDNA specific damage [e.g. PMDtools (Skoglund *et al.*, 2014)]. The results also show that even for samples as old as Ust Ishim, our method performs similarly well as for more recent samples such as Loschbour and I1583. This observation indicates that, despite the reliance on a modern reference panel, our method can work reliably on some of the oldest sequenced modern human samples. We observed a slight over-estimation (∼0.7%) of contamination on Mota, an African sample that predates the Eurasia backflow and therefore represents an ideal African reference (Llorente *et al.*, 2015). This slight bias is possibly caused by the fact that haplotype copying works less well on more diverse populations and therefore the model attributes observed mismatches to contamination. That said, this minimal upward bias does not affect the binary decision of determining whether a sample is substantially contaminated. Finally, we observed that our method can work well with a variety of contamination sources, provided that the allele frequency of the contamination source can be reasonably approximated by populations in the reference panel.

#### 3.1.2 Testing the coverage limit

**Fig. 3.**
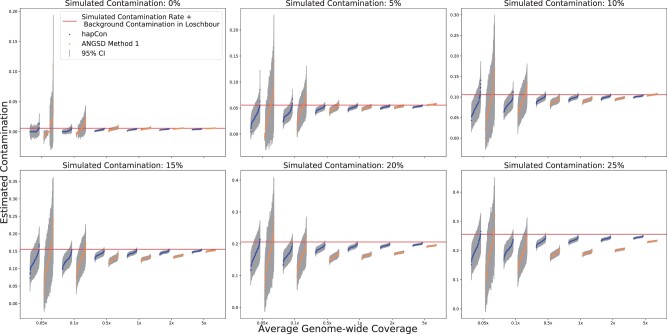
Performance comparison between ANGSD and hapCon on 1240k panel with simulated contaminated BAM files. We simulated contaminated BAM files by mixing two BAM files, using Loschbour as the endogenous source and B_French-3 as the contaminant source. We simulated contamination rate ranging from 0% to 25% with steps of 5%, and average genome-wide coverages at 5×, 2×, 1×, 0.5×, 0.1× and 0.05×. 100 replicates were created for each simulation scenario and analyzed with both hapCon and ANGSD. Contamination estimates are visualized in groups of replicates next to each other. Each · represents the estimate for one replicate, and they are ordered from low to high within each replicate group. The estimated contamination from Loschbour (0.5674%, 95% CI: 0.5669–0.5679%, estimated by ANGSD) was added to the simulated contamination rate (red horizontal line) (A color version of this figure appears in the online version of this article.)

In the next experiment, we investigated the performance of hapCon in the low coverage limit and compared it with ANGSD. For this test, we chose Loschbour as the endogenous source and B_French-3 as the contamination source. We simulated contamination rates ranging from 0% to 25% in steps of 5% and created 100 replicates for each scenario. We first tested our method using the 1240k reference panel. We estimated contamination rate on varying simulated coverages (5×, 2×, 1×, 0.5×, 0.1× and 0.05×) (Fig. 3). We found that both methods tend to under-estimate contamination at low coverage and high contamination level; however, our method has less bias than ANGSD. In addition, our method consistently yields estimates with smaller variance and narrower confidence intervals, achieving a similar level of uncertainty as ANGSD at ca. 2× lower coverage for 1240k capture data.

**Fig. 4.**
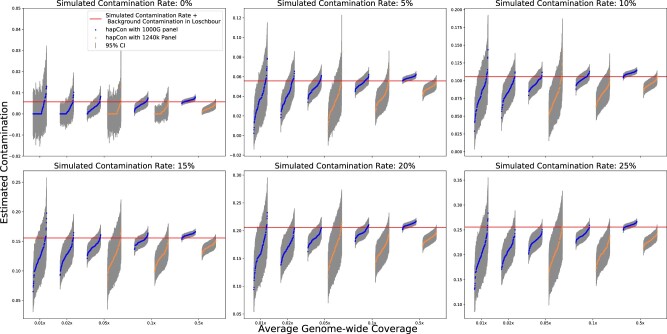
Performance comparison between two reference panels with simulated contaminated BAM files. We simulated contaminated BAM files as described in Figure 3. We simulated average genome wide coverage at 0.5×, 0.1×, 0.05×, 0.02× and 0.01×. We ran hapCon with 1000G panel on all these coverages and compared it with hapCon with 1240k on coverage 0.5×, 0.1× and 0.05×. We did not plot the results of hapCon with 1240k panel on simulated coverage 0.02× and 0.01× because the huge variance of the estimates in such low coverage regime conceals other important trends

Next, we compared our method using the 1000G and the 1240k reference haplotype panel. We estimated contamination rate on simulated coverage 0.5×, 0.1×, 0.05×, 0.02×, 0.01× using 1000G panel and 1240k panel. Our results demonstrate a substantial performance gain for estimating contamination using the 1000G panel compared with the 1240k panel. For WGS data with the 1000G panel, our method can robustly distinguish 10% contaminated samples from no contamination for as low as ∼0.02× X chromosome coverage (Fig. 4). Overall, our method achieves a similar level of uncertainty as ANGSD at ca. 10× lower coverage when using the 1000G reference panel and at ca. 2× lower coverage when using the 1240k reference panel.

### 3.2 Investigating model mis-specification

#### 3.2.1 Mis-specified contaminant allele frequency

In practice, it is often not possible to exactly specify the ancestry of the contamination. One may not have an accurate proxy for the contamination source, or a sample may be contaminated by more than one sources of contamination. Therefore, a contamination estimation method is ideally robust to mis-specified allele frequency of the contamination source.

To assess the effect of mis-specified contaminant allele frequency, we utilized synthetic BAM files simulated as described above (using Loshcbour as the endogenous source and a French individual as the contaminating source). We then estimated contamination using allele frequencies from 1000 Genome subpopulation CEU (Utah residents with Northern and Western European ancestry), FIN (Finnish in Finland), GBR (British from England and Scotland), IBS (Iberian Populations in Spain), TSI (Toscani in Italia), YRI (Yoruba in Ibadan, Nigeria), CHB (Han Chinese in Beijing, China), PEL (Peruvian in Lima, Peru). We observed that the contamination estimates obtained when using CEU, FIN, GBR, IBS, TSI allele frequencies behave very similar with little bias. These observations indicate that contamination estimates of hapCon are robust with respect to allele frequency mis-specification at the level of intracontinental genetic variation. However, estimates using CHB, PEL and in particular YRI allele frequency are substantially biased downwards (Supplementary Fig. S37). Notably, mis-specified contaminant ancestry generally does not produce upward bias and no uncontaminated sample is erroneously identified as contaminated because of mis-specified contaminant ancestry (Supplementary Fig. S36). But we observe that mis-specification at the level of intercontinental allele frequency differences introduces substantial downward biases, which may cause moderately contaminated samples to be identified as up to 50% less contaminated. A similar downward bias for substantially mis-specified contaminant ancestry was previously described for the two-consensus method (Moreno-Mayar *et al.*, 2020).

#### 3.2.2 Genetic distance between the endogenous and contaminant ancestry

When the genetic ancestry of contamination and endogenous sources are similar, the endogenous source can be closer to the allele frequencies of the specified contamination source than to the ones of the diverse reference panel. We speculated that this differential affinity creates an attraction effect, particularly at very low coverages, for the following reasons. At low coverage, most sites are covered by only one sequence and covered sites are often far apart. Without haplotype structure weighting haplotypes of the copying algorithm, the main information for estimating contamination then comes from allele frequencies. And when the contaminant allele frequency is a better fit for the endogenous source than the reference panel, there is a bias toward the contamination source. To investigate this effect, we conducted under the model simulation as described in Supplementary Note S2.1 except that we used CEU allele frequency as the contamination source. We observed that, when haplotypes simulated based on TSI (Toscani in Italia) are contaminated using CEU allele frequency, our model tends to over-estimate contamination rate at very low coverages indeed (0.05×, see Supplementary Fig. S35a and d). When reference haplotypes of African ancestry are removed, moving the reference panel allele frequencies closer to the source, the upward bias substantially decreases (Supplementary Fig. S35b and e). When using allele frequency calculated from the full reference panel for the contaminant, so that there is no allele frequency difference between the reference panel and the specified contaminant ancestry, the upward bias is completely removed (Supplementary Fig. S35c and f). However, we observed that using global allele frequency creates downward bias at low coverages in empirical aDNA data (data not shown), plausibly because of substantial allele frequency mis-specification of the contaminant when basing it on the full reference panel. As a practical compromise, we recommend using allele frequencies as closely matching the true contamination source as possible (Supplementary Figs S36 and S37) and removing highly divergent haplotypes from the reference panel.

The above-mentioned attraction effect is expected to be more pronounced when the endogenous and contaminant sources are genetically close. To explore how varying distance between endogenous and contaminant sources affects contamination estimates in practice, we conducted simulations by mixing two BAM files of individuals with varying degree of genetic distances. We fixed B_French-3 as the endogenous source, and varied the contamination source S_Sardinian-1, S_French-1, S_Hungarian-2, S_Georgian-2, S_Spanish-1, S_Korean-1 [all samples originate from the Simons Genome Diversity Project (Mallick *et al.*, 2016)]. To quantify genetic similarity, we calculated genetic distance $d_{B\_\text{French}-3,X}$ using the average hamming distance of the two samples’ genotype based on all the markers with MAF ≥ 5% in the 1000G reference panel [similar to Moreno-Mayar *et al.* (2020)]. More precisely,

(5) $$\begin{array}{c} d_{B\_\text{French}-3,X}=\frac{\sum_{s\in S}|G_{B\_\text{French}-3}(s)-G_{X}(s)|}{\left|S\right|} \end{array},$$

where *S* denotes the set of markers in the 1000G reference panel and $G_{B\_\text{French}-3}(s),G_{X}(s)$ the genotype of B_French-3, X at marker *s*, respectively. We simulated contamination rates 0%, 5% and 10% and coverages 5×, 2×, 1×, 0.5×, 0.1× and 0.05× and for each scenario we analyzed 100 independent replicates with hapCon using the 1240k reference panel. Our results indicate that genetic similarity has little effect on estimating contamination. Across all the coverages and contamination levels we tested, hapCon performs equally well regardless of the ancestry of contamination (Supplementary Figs S29, S30 and S31). We did not find any noticeable biases even in the mixed BAM simulations of a French sample contaminated with another French sample. Such little effect of the genetic distance between the contaminant and the endogenous individual has been previously reported also for the two-census model (Moreno-Mayar *et al.*, 2020). Therefore, we believe genetic similarity between the endogenous and contaminant source to not be problematic in practice. Similar performance is also observed for a Japanese sample contaminated with another Japanese sample (Supplementary Fig. S32). However, caution is warranted if the endogenous and contaminant source share IBD segments on the X chromosome, e.g. in the case of close relatives or in populations with small effective population sizes. In such cases there is a systematic downward bias of estimated contamination rate (see e.g. Supplementary Figs S33 and S34) because parts of the contaminated sequences are identical to the endogenous DNA.

### 3.3 Assessing performance using empirical aDNA data

Testing the new method on empirical aDNA data is an important validation step because some complexity of empirical aDNA data is potentially not accurately reflected in simulation models. Therefore, we performed a series of experiments down-sampling empirical aDNA data, and compared ANGSD and hapCon on various data across a wide range of ancestry, age, coverage and data type.

#### 3.3.1 Down-sampling previously published 1240k and WGS data

First, we down-sampled published BAM files from previous aDNA studies. For 1240k data, we explored two male individuals from Sardinia, SUA001 (1411-1228 calBCE, 1.02× chrX coverage on 1240k SNP sites) and SUA002 (2274-2032 calBCE, 0.64× chrX coverage on those sites) (Marcus *et al.*, 2020). We chose those two because ANGSD estimates SUA001 to be substantially contaminated (10.45%, 95% CI: 9.56–11.34%) and SUA002 to be only slightly contaminated (0.38%, 95% CI: 0.072–0.69%).

**Fig. 5.**
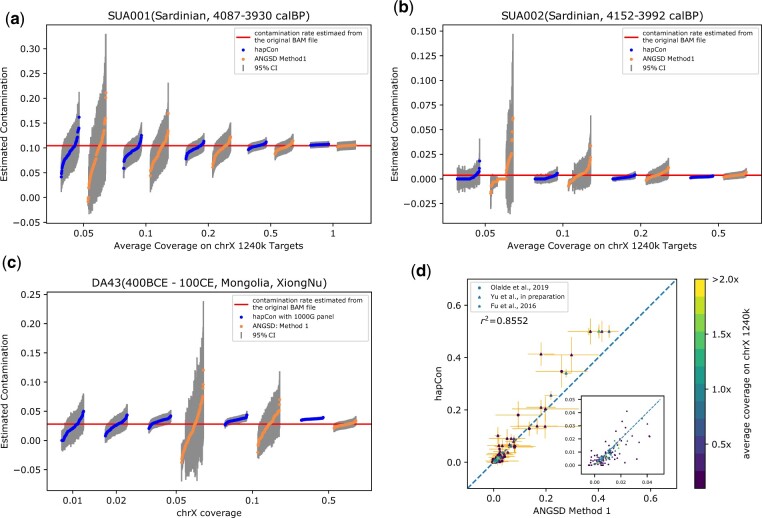
Assessing performance on empirical aDNA Data. (**a, b**) We performed downsampling experiments on 1240k data of two Sardinian samples, SUA001 and SUA002, both from Marcus *et al.* (2020). The original BAM files were down-sampled to various coverages with 100 independent replicates for each coverage. (a) Comparison between our method and ANGSD on SUA001, estimated to be 10.45% (95% CI: 9.56–11.34%) contaminated by ANGSD (on full data, visualized by the horizontal red line). (b) Comparison between our method and ANGSD on SUA002, estimated to be 0.38% (95% CI: 0.072–0.69%) contaminated by ANGSD (on full data, visualized by the horizontal red line). (**c**) We down-sampled WGS data of DA43, XiongNu, Mongolia from de Barros Damgaard *et al.* (2018). The original BAM file for DA43 was down-sampled to various coverages 0.01–0.5×, with 100 independent replicates for each target coverage. We only visualized ANGSD’s results on 0.05×, 0.1×, 0.5× because its estimates at coverage lower than 0.05× were highly variable. DA43 is estimated to be 2.83 % (95% CI: 2.35–3.31%) contaminated by ANGSD (on full data, visualized by the horizontal red line). (**d**) We compared our new method and ANGSD on 1240k aDNA data of 89 samples from the Iberian Peninsula and of 66 Eurasian hunter-gatherers. The true contamination rate is unknown. No down-sampling was performed and all individuals (dots) are color coded by the average coverage on 1240k SNPs on chromosome X. The inlet visualizes a zoom-in into [0,0.05]×[0,0.05]. A similar figure that only shows the Eurasian hunter-gatherers is available in Supplementary Figure S13

For each target coverage, we independently down-sampled 100 replicates (Fig. 5a and b). For the highly contaminated sample (SUA001) at coverage 0.05×, our method identifies 98 replicates as having substantial contamination (here defined as >5%), while ANGSD identifies only 80 as having substantial contamination. For the minimally contaminated sample (SUA002) at coverage ∼0.05×, our method identifies all 100 replicates as minimally contaminated (<5%), while ANGSD’s estimate ranges from 0% to greater than 5%, falsely identifying two replicates as having substantial contamination. For 0.1× coverage, our new method can robustly distinguish minimally and substantially contaminated samples—all the down-sampled SUA001 replicates have contamination estimates greater than 5%, and all SUA002 replicates have contamination estimates less than 5%. Together, these down-sampling experiments showed that hapCon can robustly identify substantial contamination in empirical 1240k aDNA data for coverage as low as 0.1×.

For comparison, we applied the two-consensus method (Moreno-Mayar *et al.*, 2020) and contamLD (Nakatsuka *et al.*, 2020) to these two Sardinian samples. We found that the two-consensus method performs overall similarly to ANGSD, but on some 0.05× coverage replicates much worse (Supplementary Fig. S26). We also observed that contamLD performs similarly well as ANGSD at high coverages; however, it suffers from much more substantial biases than ANGSD at lower coverages (Supplementary Fig. S27). Our simulations also showed that contamLD is either more biased or has higher variance than our method at low coverages (Supplementary Fig. S28). Therefore, we focused the overall analysis on comparison between our new method and ANGSD, which is also currently most widely used method for male contamination estimation.

For down-sampling experiments of WGS data, we used a XiongNu sample DA43 (Mongolia, 400BCE-100CE, 0.83× chrX coverage) (de Barros Damgaard *et al.*, 2018), which is estimated to be 2.83% (95% CI: 2.35–3.31%) contaminated by ANGSD. Since this sample is WGS data, we could use the 1000G reference panel. We tested our method’s performance on coverage 0.01×, 0.02×, 0.05×, 0.1×, 0.5× and compared to ANGSD (Fig. 5c). Our results show that hapCon yields reliable estimates down to about 0.02× coverage on the X chromosome for WGS data, achieving similar confidence intervals at 10× lower coverage than ANGSD. This performance gain is similar to that observed on the tests on mixed BAM files (Fig. 4).

#### 3.3.2 Comparing hapCon and ANGSD on published aDNA data

To systematically compare hapCon and ANGSD estimates on empirical data, we applied both methods to 1240k aDNA data including a wide range of coverages and contamination rates. We selected all 89 ancient males from Olalde *et al.* (2019) that have coverage greater than 0.05× on the X chromosome, all of which are from the Iberian Peninsula and date to within the past 8000 years. To test our method on even older samples which are genetically more distant from the modern reference panel, we additionally tested both methods on 60 male Eurasian hunter-gatherer samples (Yu *et al.*, in preparation) and six male samples with at least 0.05× coverage on chrX from Fu *et al.* (2016). We found that estimates from hapCon and ANGSD are highly concordant on the full sample set ($r^{2}=0.8552$), and for 145 out of 155 samples hapCon provides smaller confidence intervals (Fig. 5d). For the 124 samples with contamination rate estimated to be <5% by both methods, the estimate of hapCon is higher than that of ANGSD on 41 samples, and lower on the remaining 83 samples, indicating that both methods give overall similar estimates when the contamination rate is low. In contrast, for all samples with contamination rates >20%, hapCon generally estimates higher contamination than ANGSD. We note that our simulation experiments have shown that ANGSD has substantially more downward biases than our method in the high contamination regime (Fig. 2), thus the higher estimates of hapCon are likely closer to the true contamination rate. In any case, samples with contamination rate substantially greater than 10% are excluded from downstream analysis in practice.

Although our method works equally well on Upper Paleolithic and Mesolithic Eurasian hunter-gatherers as on more recent samples and for a wide range of global ancestries (Fig. 2), we note that caution is warranted when working with data containing deeply diverged haplotype not captured well by the 1000 Genome reference panel. For instance, for some south and central African forager data, our method may overestimate contamination (Supplementary Note S4.2).

## 4 Discussion

We have presented a new approach to estimate aDNA contamination in male modern humans based on a Li&Stephens haplotype copying model and implemented it in a software package (hapCon). The Li&Stephens model, widely used in population genomics, makes use of haplotype structure and linkage disequilibrium information, and constitutes a central part of many modern phasing and imputation algorithms (Browning *et al.*, 2021, e.g. Delaneau *et al.*, 2019; Loh *et al.*, 2016; Rubinacci *et al.*, 2021). Similarly, our method implicitly imputes the endogenous genotype using reference haplotypes, and thus can utilize sites covered by only one sequence, which, to our knowledge, cannot be effectively utilized by any other male X chromosome-based method. Tests on simulated and down-sampled empirical aDNA data showed that the new approach substantially improves power to estimate contamination, particularly in the low coverage regime. Across coverage levels, hapCon consistently yields estimates with lower variance and narrower confidence intervals than ANGSD and the two-consensus approach described in (Moreno-Mayar *et al.*, 2020). The most substantial gains are achieved for low-coverage WGS data. We found that hapCon provides robust contamination estimates for 1240k capture data with as low as 0.1× coverage and for WGS data with as low as 0.02× coverage on the male X chromosome, substantially extending the limits of ANGSD or the two-consensus approach. We explored various sources of model mis-specifications, including sequencing error, post-mortem damage, haplotype copying jump rate, distance to reference panel and mis-specified contaminant allele frequencies. These experiments showed that hapCon is robust with respect to reasonable mis-specifications. Moreover, we observed that contamination estimates do not depend on genetic distances between the endogenous and contaminant ancestry.

There are several limitations of our new approach. Haplotype copying substantially improves the power; however, it requires that the true endogenous haplotype can be modeled well as a mosaic of modern haplotypes. Deeply diverged human lineages such as Neanderthals and Denisovans are outside the range of this copying model. In such cases, one should consider using ANGSD or other methods not relying on a haplotype reference panel (e.g. Peter, 2020). Having that said, our experiments demonstrated that our method works on Ust Ishim (46 880–43 210 calBP), one of the oldest sequenced modern humans, similarly well as on other more recent samples. Additionally, we have tested our method on Paleolithic and Mesolithic hunter-gatherers and found good correlations between estimates from our method and that from ANGSD, indicating that the new haplotype copying approach in principle works for most modern human aDNA. Another issue that we identified is a moderate upward bias at low coverage when the allele frequency of the specified contaminant source is substantially closer to the endogenous source than the overall reference panel, but using an Out-of-Africa haplotype reference panel partially alleviates this bias (Supplementary Fig. S35). Finally, our results showed that the specified contaminant allele frequencies should remain within continental genetic variation of the true contamination source, otherwise contamination estimates can become substantially downward biased. If there is no prior information about the contamination source or the sample has been contaminated by several sources from different continental ancestries, our method may yield substantially biased results, in particular for highly contaminated samples.

Beyond application to the naturally haploid male X chromosome, we envision our haplotype copying approach to be useful for estimating contamination for female samples with long runs of homozygosity (ROH), as such regions are effectively haploid. Previous studies have identified extensive ROH in almost all paleolithic hunter-gatherers (Ringbauer *et al.*, 2021) or in populations with small effective size, such as the pre-contact Caribbean (Fernandes *et al.*, 2021). However, we note that contamination interferes with identifying ROH, particularly in the low coverage regime. Future work could establish robust approaches to identify ROH for substantially contaminated data, and the software presented here can then be straightforwardly extended for estimating contamination on ROH.

Utilizing reference panels that are larger and better represent diverse ancestries could extend the application range of our method. In particular, the genetic diversity of the African continent is underrepresented in the current reference panel based on 1000 Genomes dataset; we found that our haplotype copying approach using this default reference panel suffers from biases when modeling samples containing central and southern African ancestry (Supplementary Note S4.2). The generation of more diverse haplotype reference panels is currently on the way (Choudhury *et al.*, 2020; Fatumo *et al.*, 2022), and those panels could substantially improve the performance of our method.

Another promising extension is to utilize a full diploid Li&Stephens copying model as in Lunter (2019) to directly estimate contamination on autosomes, thereby enabling estimating autosomal contamination in female samples. This approach would be closely related to imputing diploid genotype, which often similarly relies on the diploid Li&Stephens model. A particular challenge of such a diploid approach is that diploid imputation is much more challenging than the haploid imputation. In particular in the low coverage regime (<0.5×), that constitutes the majority of aDNA data, diploid imputation accuracy is limited and could produce substantial biases that interfere with estimating contamination (Ausmees *et al.*, 2022; Hui *et al.*, 2020; Rubinacci *et al.*, 2021).

## Supplementary Material

btac390_Supplementary_Data
