# Supplementary material for: hapCon: estimating contamination of ancient genomes by copying from reference haplotypes
Source: Bioinformatics. 2022 Jun 13;38(15):3768–77. doi: 10.1093/bioinformatics/btac390 (PMC9344841; doi:10.1093/bioinformatics/btac390)
Supplement: btac390_Supplementary_Data [file btac390_supplementary_data.pdf]

Supplementary Notes

Yilei Huang[1,†] and Harald Ringbauer[1,†]

[1]Department of Archaeogenetics, Max Planck Institute for Evolutionary Anthropology, Leipzig, Germany

[†]Corresponding author – Email: yilei_huang@eva.mpg.de, harald_ringbauer@eva.mpg.de

May 2022

1 Calculating Confidence Interval via 14.7% Likelihood Region

Our model is defined only for contamination within range 0 to 1. Therefore, when the maximum likelihood estimate $\hat{c}$ for the contamination rate c is 0 the first derivative there might not be zero. In this case, we approximate the likelihood curve using quadratic interpolation. Specifically, let $l(x)$ be the likelihood of the model at contamination rate $c = x$, then, for x close enough to 0, we have

$$l(x) = l(0) + xl'(0) + \frac{x^2}{2}l''(0) + o(x^2)$$

Using this approximation around 0 and ignoring the second order term $o(x^2)$, we then apply Newton's method to find the solution of $l(x) - 1.92 = 0$. This is the so-called 14.7% likelihood region approach. Briefly, a p% likelihood region is defined as the set $\{\theta \in \Theta : \frac{L(\theta)}{L(\theta_{MLE})} \geq \frac{p}{100}\}$. When $p = 14.7$, then

$$P\left(\{\theta \in \Theta : \frac{L(\theta)}{L(\theta_{MLE})} \geq \frac{14.7}{100}\}\right) = P(\theta \in \Theta : -2(l(\theta) - l(\theta_{MLE})) \leq 3.835)$$
$$\approx P(\chi_1^2 \leq 3.835) = 0.9498$$

The log of likelihood ratio $-2(l(\theta) - l(\theta_{MLE}))$ asymptotically approaches χ_1^2 as a result of Wilk's theorem. Therefore, the 14.7% likelihood region approximates the 95% confidence interval.

Note that $\frac{L(\theta)}{L(\theta_{MLE})} >= 0.147$ is equivalent to $log(L(\theta)) - log(L(\theta_{MLE})) \geq -1.92$. For full details, we refer to [Rossi, 2018, Definition 5.11].

2 Additional Simulations

Several parameters in our model need to be set, but the ideal values for those can often not be exactly determined for each use case. To assess the general robustness of contamination estimates, we performed under the model simulations, where we first generate data as assumed in our generative HMM model. Single parameters are altered around their default values to test our model under a variety of model mis-specifications, as described below.

2.1 Default Simulation Settings

Here we describe the default setting for the simulations. To create genotype data, we first copy haplotype blocks with miscoyping rate $1e^{-3}$ from TSI (Tuscany, Italy) haplotypes in the 1000Genome Dataset (Phase 3, [Consortium et al., 2015]). A copied haplotype block is chosen randomly with equal probability from all reference haplotypes, with each copied segment having length drawn from an exponential distribution with mean length 1/3 centimorgan. At the end of each copied block, a new reference haplotype is chosen and copied from. Having simulated a mosaic of reference haplotypes, for each marker i in the 1240k panel we then draw read counts from a Poisson distribution with mean equal to the target coverage multiplied by a weighing factor λ_i. This weighing factor models that in 1240k capture data some sites are systematically more likely to be covered than others. We obtain this weighting factor λ_i by comparing site coverage to genome-wide average coverages in all male samples in Olalde et al. [2019]. Contaminant sequences are then drawn according to the global allele frequency in the 1000Genome dataset. To simulate sequencing genotype error, we flipped the genotype of every sequence to the other allele with probability $1e^{-2}$. As reference panel for inference, we used all 1000Genome haplotypes excluding TSI samples.

Using this above described default simulation scheme, we first simulated average coverage 20x, 10x, 5x, 2x, 1x, 0.5x, 0.1x and 0.05x with contamination rate up to 25% and analyzed the simulated read counts with our implementation of hapCon. Reassuringly, we obtained accurate

estimates with little bias for all coverages and contamination rates tested here (Fig. S1), thereby confirming the correctness of our implementation in under the model simulations.

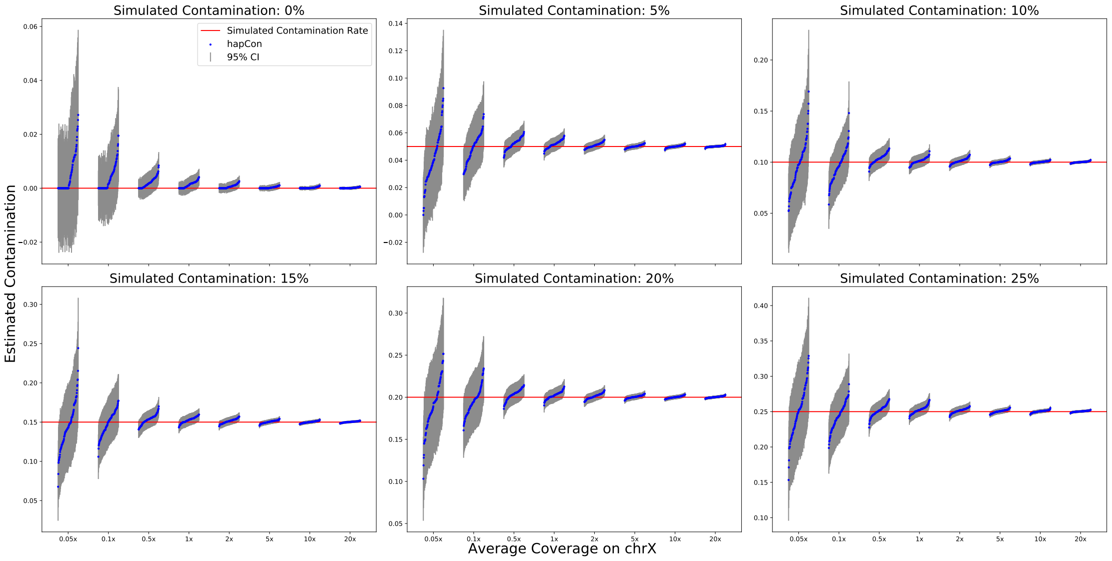

Figure S1: **Performance on Simulated Read Counts Data at Various Coverages and Contamination Rates**. Data simulated under the model as described in the text.

2.2 Down-sampling Simulated Read Counts to Pseudohaploid Data

In this simulation scenario, we randomly sampled one sequence for each marker covered by at least one sequence. This procedure simulates how the so-called pseudohaploid data is generated from aDNA data. We note that, even though each site is only covered by one sequence, a high coverage sample will have more sites covered and therefore still contain more information than a low coverage sample. Our results show that our method produces robust contamination estimates even for such pseudohaploid data (Fig. S2), which none of the existing male X chromosome contamination estimation tools can do. Compared with Fig. S1 where the full read counts data is used, the estimates obtained from pseudohaploid data only have minimal upward bias. In practice, we recommend using our method on read counts directly generated from a BAM file to make full use of all information, but this simulation of pseudohaploid data demonstrates the power of utilizing haplotype structure for estimating contamination.

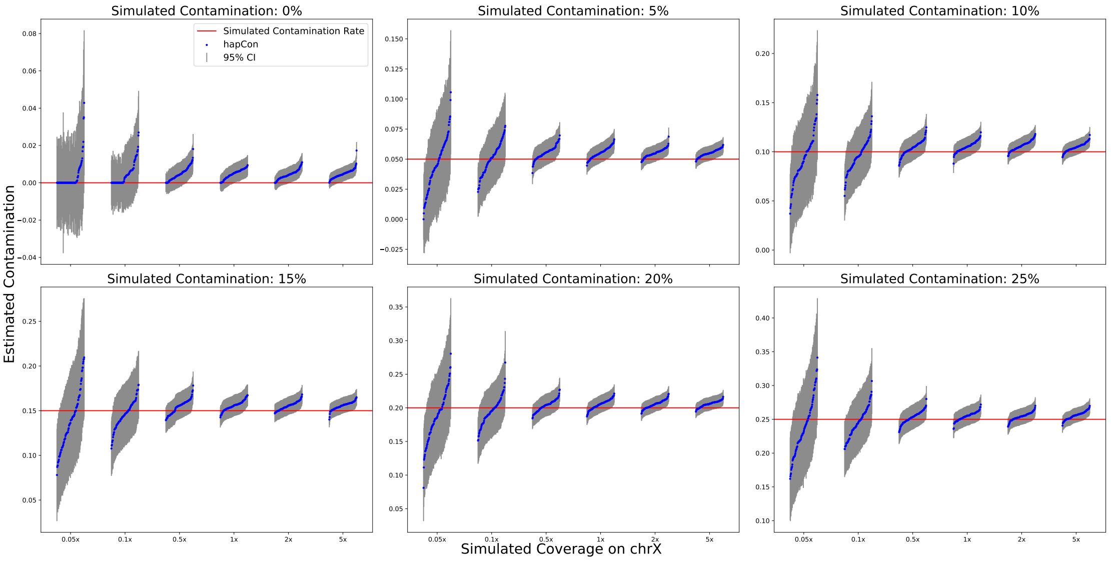

Figure S2: **Performance on Simulated Pseudohaploid Data at Various Coverages**

2.3 Model Mis-specification

2.3.1 Mis-specified Error Rate ϵ_g

In practice, we estimate error rate ϵ_g from discordant sequences at sites flanking to the sites contained in the reference panel, as in Rasmussen et al. [2011], Moreno-Mayar et al. [2020]. These sites are expected to be fixed; therefore, any discordant sequences reflect only sequencing error/mis-mappings/aDNA damage and not contamination. Suppose there are a total of L non-polymorphic sites in the reference panel and let M_l, m_l denote the count of major and minor sequences at site l respectively. Then we estimate error ϵ_g by

$$\epsilon_g = \frac{\sum_{l=1}^{L} m_l}{\sum_{l=1}^{L} M_l + m_l}$$

This implicitly assumes that sites adjacent to markers in the reference panel are fixed, including the contamination source. We use four adjacent sites on either side of the polymorphic marker to estimate this error rate.

When taking read counts from a BAM file, we only count sequences matching either the reference or alternative allele in the reference panel. Sequences that match neither of these two alleles are discarded. Therefore, we set $\frac{\epsilon_g}{3}$ as the error parameter in our HMM model since ϵ_g as

estimated above captures the possibility of misreading a base to all three other bases. We note that this is an average approximation; for example, post-mortem damage preferentially produce C→T and G→A mismatches. However, explicit modeling of biased error rates is complex and case-dependent, here we aim for a simple model that empirically approximates a wide range of application scenarios.

To evaluate how mis-specified error rate affects our method, we simulated 10% contamination as described in Section 2.1 except that we varied the simulated error ranging from $1e^{-4}$ to $1e^{-2}$, evenly spaced on a log scale. We then estimated contamination rate when setting $\epsilon_g = 1e^{-3}$ for all simulated samples. This value is within the typical range of error rate estimates from empirical aDNA data.

We observe moderate upward bias of contamination estimates when the specified error rate is substantially below the true error rate (Fig. S3). A plausible intuitive reason for this bias is that if the mismatches observed cannot be fully explained by error the method attributes mismatches to contamination. Importantly, we observe that bias remains on the same order of magnitude as the mis-specification of the error rate. In empirical aDNA studies the error rate is usually on the order of $1e^{-3}$, whereas one wishes to estimate contamination on the order of $1e^{-2}$ or higher. Therefore, mis-specified error rate should not introduce relevant biases in most empirical analyses.

2.3.2 Mis-specified Haplotype Copying Error Rate ϵ_r

Some events such as mutations, gene conversions, or errors in the reference panel can cause sporadic genotype mismatches between the copied haplotype and the endogenous haplotype of interest. Therefore we use a copying error rate to model such mismatches. Following other methods that use Li&Stephens copying model[Rubinacci et al., 2021, Loh et al., 2016], we set $1e^{-3}$ as the default copying error rate. To evaluate the effect of mis-specified copying error rate, we simulated 10% contamination as described in Section 2.1 except that we varied the miscopying rate from $1e^{-4}$ to $1e^{-2}$, evenly spaced on a log scale. We then estimated contamination rates when setting $\epsilon_r = 1e^{-3}$ for all simulated samples.

The results indicate that the contamination estimate is robust to mis-specified copying error rate (Fig. S4). Even in cases where the miscopying rate is $1e - 2$, one magnitude higher than

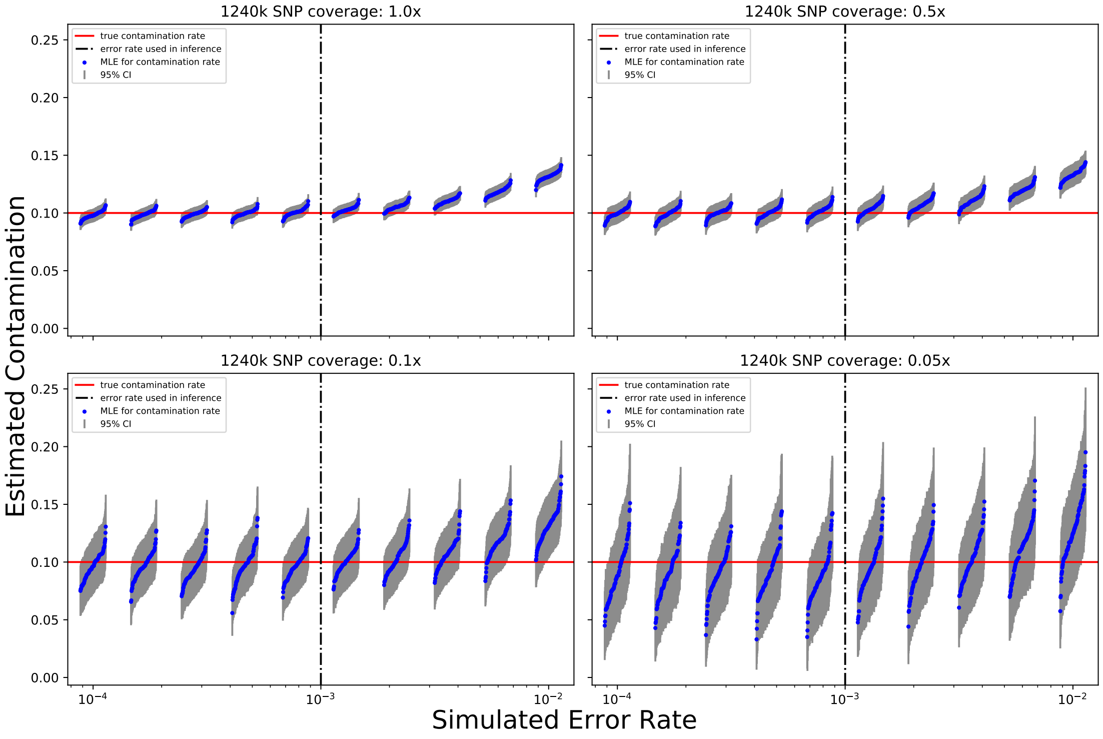

Figure S3: **Effect of Mis-specified Error Rate at Various Coverages**

assumed, upward bias remains small. Similar to the case of mis-specified error rate discussed above, we observe that bias remains on the same order of magnitude as the mis-specification of mis-copying rate. In empirical data the error rate is usually on the order of $1e^{-3} - 1e^{-4}$, depending on the genetic distance between the endogenous haplotype and the modern haplotypes, whereas one wishes to estimate contamination on the order of $1e^{-2}$ or higher. Therefore, mis-specified mis-copying rate should not introduce substantial biases in empirical analyses.

2.3.3 Mis-specified Haplotype Copying Jump Rate

The haplotype copying jump rate models the rate of jumping to a different haplotype to copy from. Following Ringbauer et al. [2021], who described that $\rho = 300$ yields good performance for most modern human ancient DNA data when using Li&Stephens model for inferring ROH, we set $\rho = 300$ as the default value. We then assessed whether our model is robust with respect to a mis-specified jump rate. To do so, we simulated 10% contamination as described in Sec-

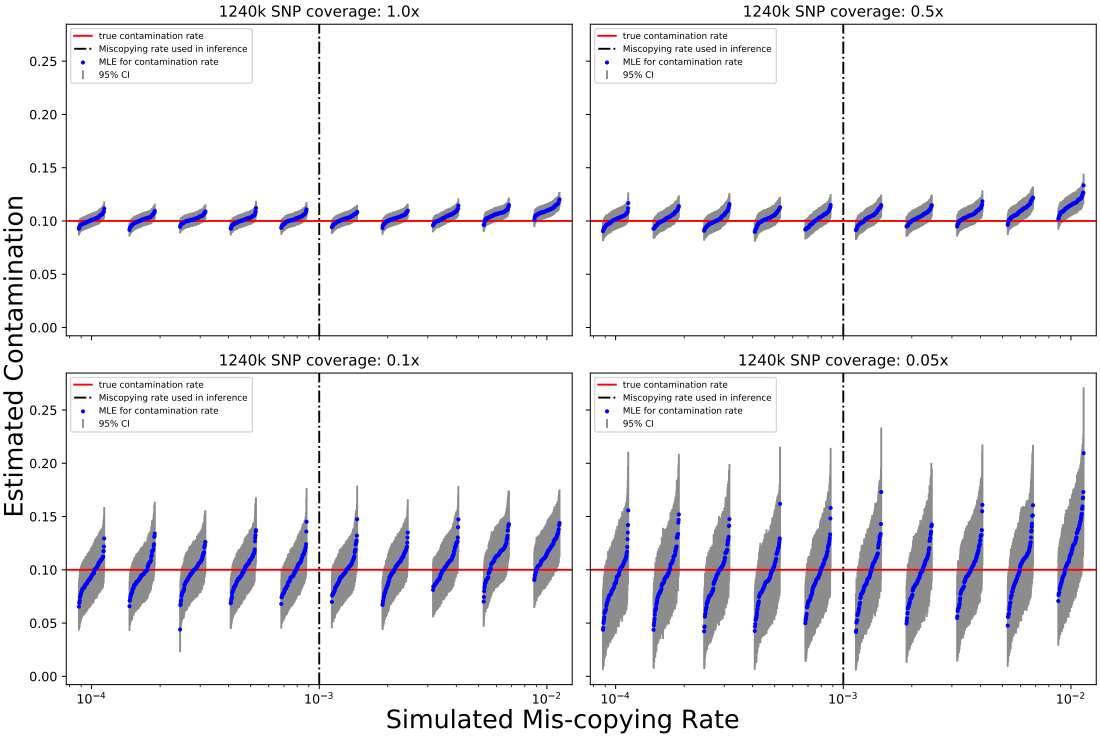

Figure S4: **Mis-specified Haplotype Copying Error Rate at Various Coverages.**

tion 2.1 except that we varied the jump rate from 100 to 1000, evenly spaced on a log scale. We then estimated contamination rate when setting the jump rate to $\rho = 300$.

The results show that the estimated contamination remains unbiased for a wide range of simulated jump rates when using the default value $\rho = 300$ (Fig. S5). Only at high jump rates close to $\rho' = 1000$, we observe some upward bias. This upward bias remains minimal at relatively high coverage ($\sim$ 1x), only at lower coverage we observe moderate upward bias ($\sim$0.5x or lower, Fig. S5b,c,d). That said, previous work showed that the estimated maximum likelihood haplotype copying jump rate never exceeds 800 in 344 ancient male X chromosomes examined, with the majority of them within range 300-600 [Biddanda et al., 2021, Fig. 7]. Therefore, we believe that $\rho = 300$ is a suitable default setting that performs well on the majority of ancient DNA data.

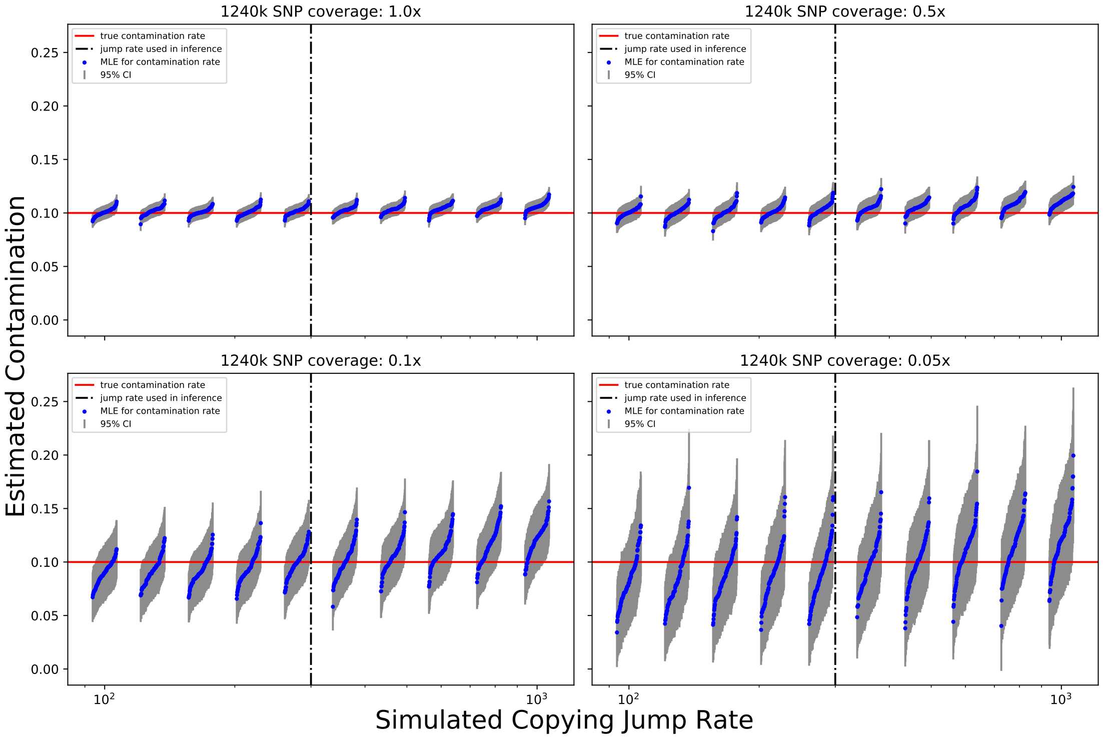

Figure S5: **Effect of Mis-specified Haplotype Copying Jump Rate at Various Coverages.**

2.3.4 Effects of Post-mortem Damage on Contamination Estimation

Once an organism dies, its DNA begins degrading. In particular, the deamination process turns Cytosine into Uracil, which is then read as Thymine by the sequencing machine. This characteristic C→T damage pattern is widely used to verify the authenticity of aDNA module in sequencing libraries. The extent of this post-mortem damage is dependent upon a variety of factors, including sample ages, preservation conditions, and library preparations. Half-UDG and full-UDG treatment have been developed to turn Uracil back to Cytosine to reduce the level of post-mortem damage. Due to this unique deamination process, aDNA has elevated level of C→T error rates in the forward strand (and G→A error rates on the reverse strand for double-strand libraries); therefore, this constitutes a model mis-specification for our error rate where we assumed all twelve possible transitions and transversions are equally likely. In this section, we simulated various levels of post-mortem damage to evaluate how it affects our method's contamination estimates.

We estimated empirical damage rates along the aDNA sequences using mapDamage2.0 [Jónsson et al., 2013] on LaBrana (5982-5741 calBCE, double stranded library) [Lazaridis et al., 2014], Zlatý kůň (450,00BP or older, sample age estimated from Neanderthal introgression segment length, single stranded library) [Prüfer et al., 2021] and Bacho Kiro CC7-335 (45,930-42,580 cal BP, single stranded library) [Hajdinjak et al., 2021]. All three samples are non-UDG treated, therefore all C→T transitions accumulated over time are preserved during library preparation. The estimated misincorporation rates are summarized in Fig. S6. Only LaBrana is prepared using the double stranded protocol, therefore only this sample shows the G→A pattern at the 3′ end; for completeness, however, we visualized 3′ G→A rates for all three samples.

We used B_French-3 as the endogenous source and S_French-1 as the contaminant. Both samples are from Simons Genome Diversity Project [Mallick et al., 2016]. We used Gargammel [Renaud et al., 2017] to add post-mortem damage to the sequences from the endogenous source using empirical C→T transition rates estimated from the BAM files of the three samples described above. We then re-aligned the damaged sequences to the reference genome hs37d5 with BWA 0.7.17-r1198g using the parameter setting -n 0.01, -o 2, and -l 16500 commonly used for aDNA data. We down-sampled the BAM file of B_French-3 and S_French-1 and mixed them to create desired genome wide coverages and contamination rates.

Our results indicate that, post-mortem damage leads to little bias for our contamination estimates (Fig. S7, S8, S9), even for data without any UDG treatment and with 5′ terminal C→T transition rate as high as 36.6% (e.g, in Bacho Kiro CC7-335).

We also examined how different levels of post-mortem damage affect the parameter ϵ_g, which is the error rate per aligned aDNA sequence base as described in the main text. This error rate is intended to model several sources of errors, including sequencing error, post-mortem damage and mismappings. Therefore, the ϵ_g inferred from sites adjacent to the polymorphic sites increase with increasing levels of aDNA damage. Indeed, as shown in Fig.S10,S11 and S12, the estimated ϵ_g increases as the damage level increases. We note that in general the average ϵ_g estimated at low coverage is the same as the average ϵ_g estimated at higher coverage. Specifically, we use a short red horizontal line to indicate the ϵ_g averaged over 100 replicates at 5x coverage for each of the damage levels, and at low coverage we observe that the blue dots center around the horizontal red bar, but with higher variance at low coverages as expected.

Comparing the estimated ϵ_g at different contamination rates for each of the damage levels, we observe that it remains stable across contamination rates (see Table.S1), indicating that our assumption that the sites adjacent to the polymorphic sites are fixed (and thus also not altered by contamination) is reasonably valid.

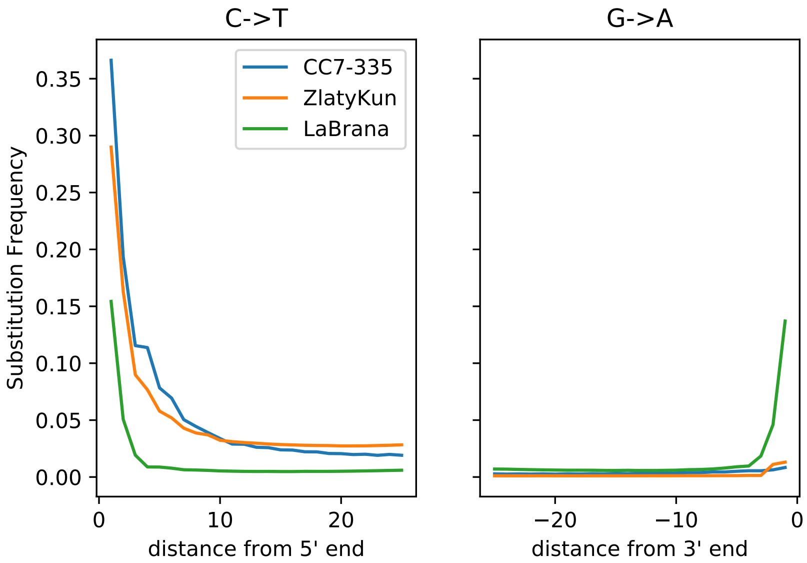

Figure S6: **Estimated post-mortem damage on LaBrana, Zlatý kůň and Bacho Kiro CC7-335** Estimated C→T and G→A substitution rates along the aDNA sequences of LaBrana, Zlatý kůň and Bacho Kiro CC7-335.

Table S1: **Estimated ϵ_g (average over 100 replicates) at Different Contamination Rate for Different Damage Patterns at 5x coverage**

Damage Pattern \ Contamination Rate	0%	5%	10%
No Damage	0.000190	0.000214	0.000238
LaBrana	0.00318	0.00306	0.00294
Zlatý kůň	0.00868	0.00831	0.00793
Bacho Kiro CC7-335	0.0105	0.010	0.00960

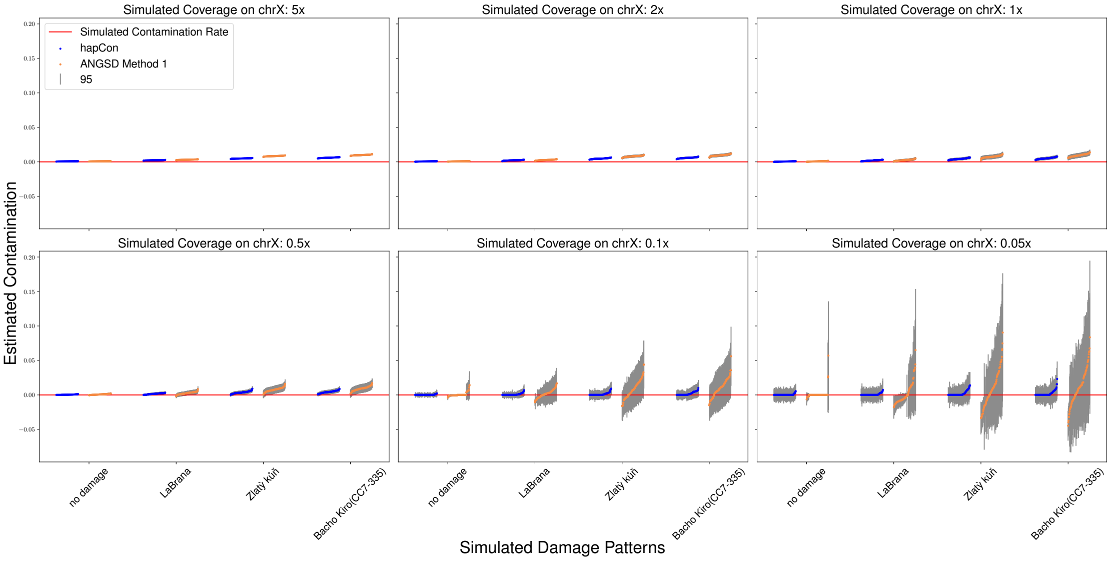

Figure S7: **Effects of Post-mortem Damage on Contamination Estimation for Simulated 0% Contamination** We simulated damaged sequences as described above and visualized results of ANGSD and hapCon with 1240k panel.

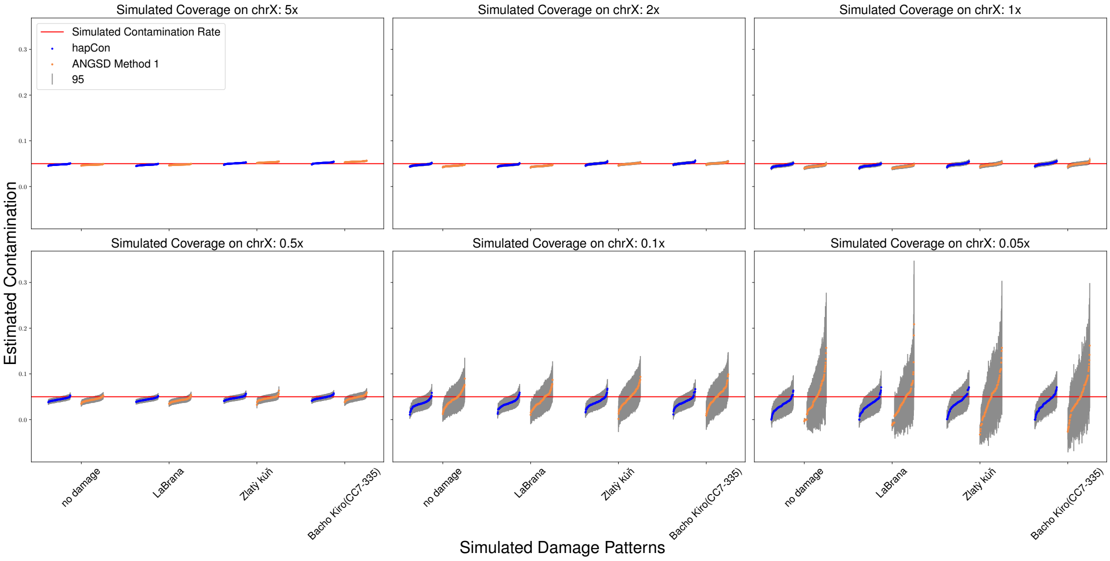

Figure S8: **Effects of Post-mortem Damage on Contamination Estimation for Simulated 5% Contamination** Same as Fig.S7 but with 5% simulated contamination.

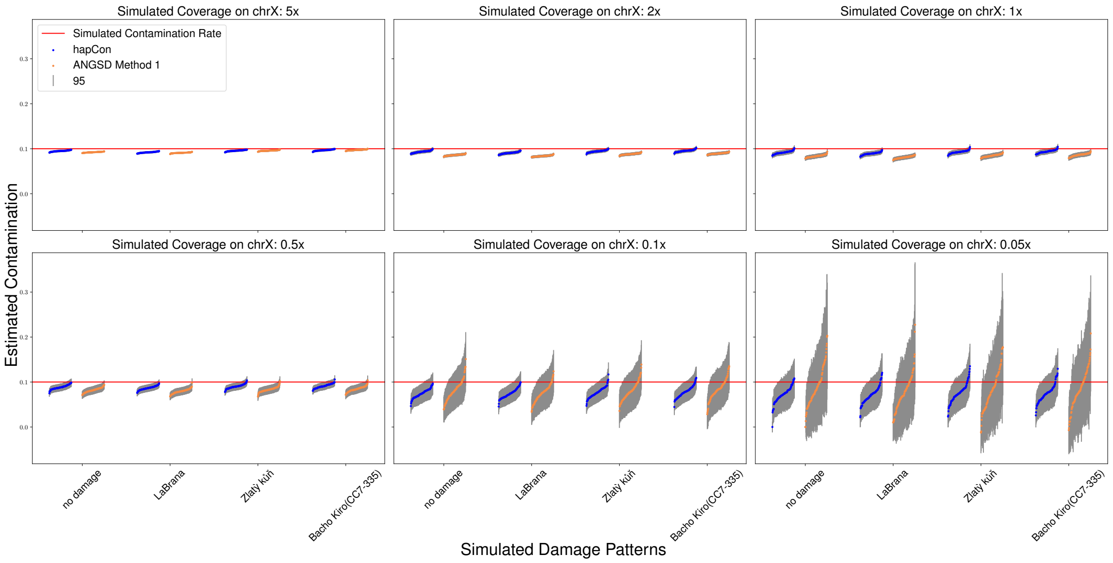

Figure S9: **Effects of Post-mortem Damage on Contamination Estimation for Simulated 10% Contamination**Same as Fig.S7 but with 10% simulated contamination.

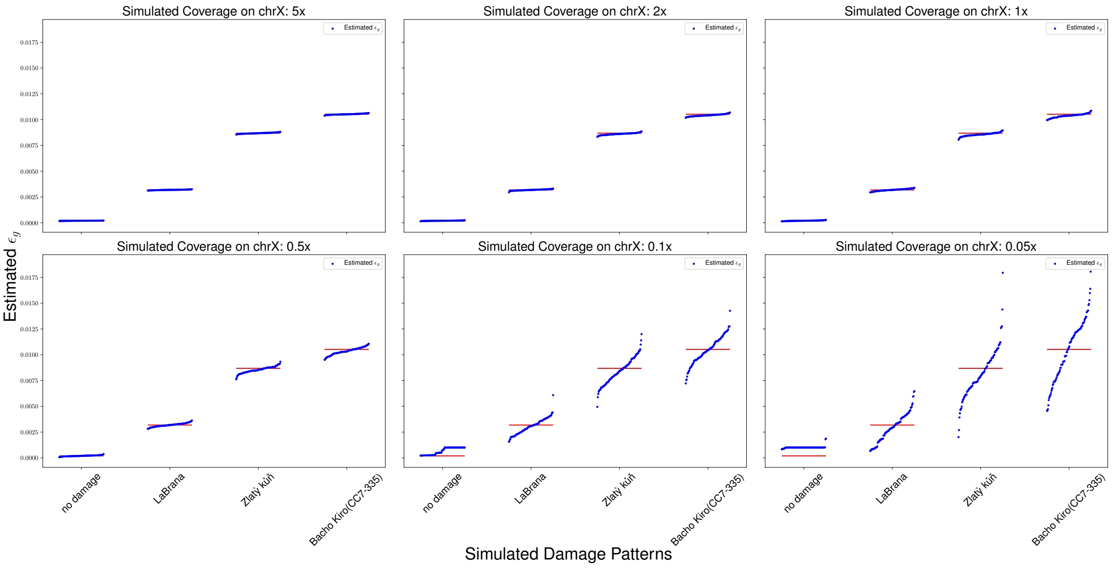

Figure S10: **Effects of Post-mortem Damage on Estimated ϵ_g for Simulated 0% Contamination** We visualized the effects of different levels of post-mortem damage on the estimated ϵ_g. The red horizontal bar represents the estimated ϵ_g at simulated 5x coverage for each of the damage levels averaged over 100 replicates.

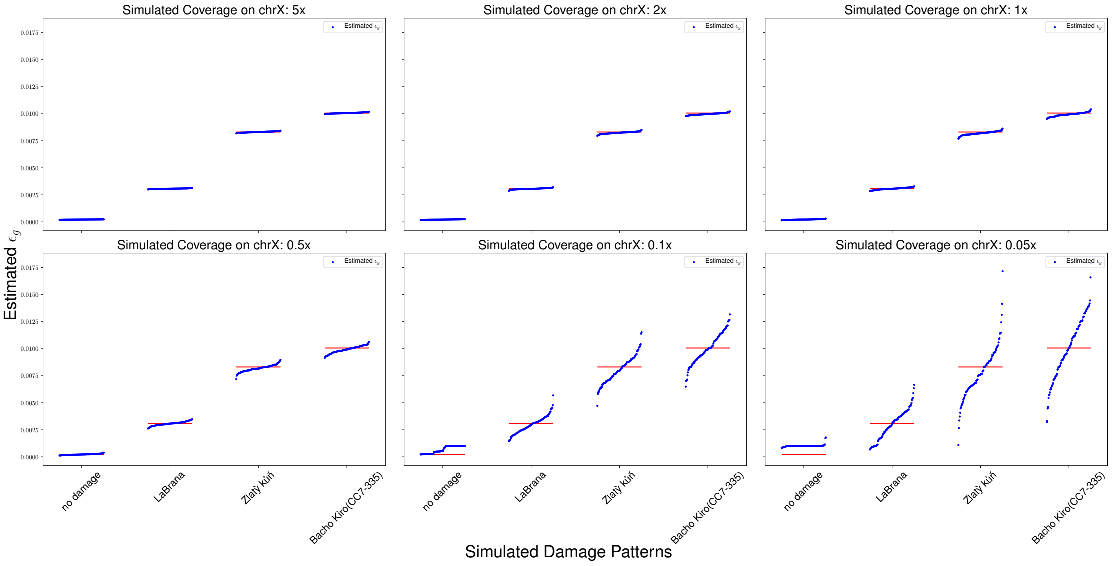

Figure S11: **Effects of Post-mortem Damage on Estimated ϵ_g for Simulated 5% Contamination** Same as Fig.S10, but with 5% simulated contamination.

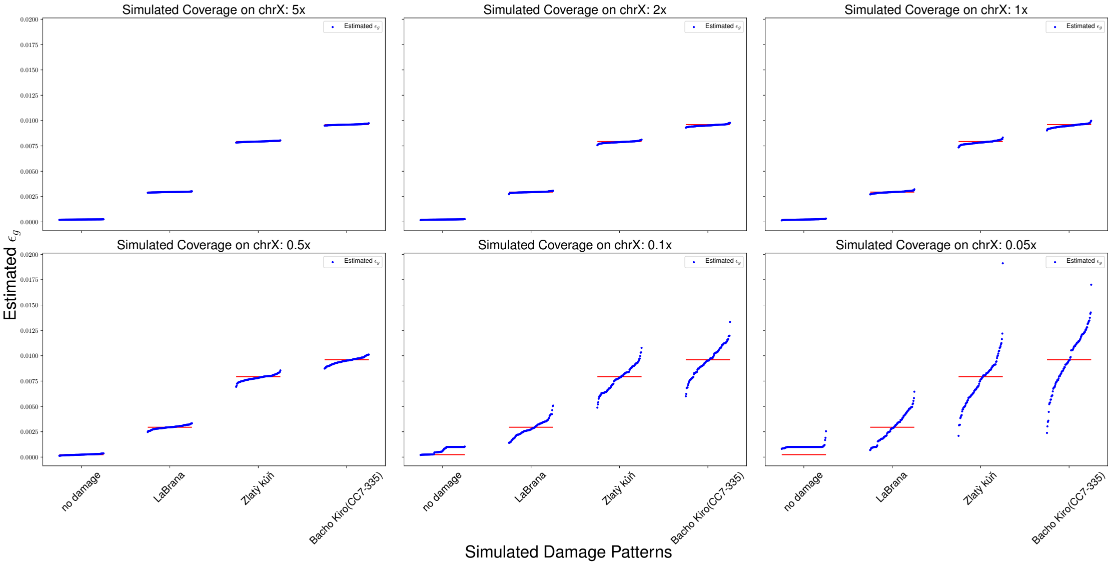

Figure S12: **Effects of Post-mortem Damage on Estimated ϵ_g for Simulated 10% Contamination** Same as Fig.S10, but with 10% simulated contamination.

3 A List of Software&Python Packages Used in This Work

- ANGSD 0.934
- samtools 1.13
- Python 3.8.10
- Numpy 1.17.4
- Scipy 1.4.1
- Numdifftools 0.9.39
- h5py 3.6.0
- bwa 0.7.17-r1198
- Gargammel
- mapDamage2.0
- contamLD

4 Testing on empirical Hunter-gatherer aDNA

Our model relies on the assumption that most ancient genomes can be modeled by a mosaic of modern haplotypes, and hunter-gatherer groups represent some of the most diverged genetic ancestry currently available for modern human aDNA research. Therefore, we performed additional test using a variety of hunter-gatherer samples to show that hapCon works well on Eurasia and African hunter-gatherer ancestry (except for central and southern African forager ancestry).

4.1 Eurasian Hunter-gatherer

First, we compiled a set of 66 male Eurasian hunter-gatherer samples, 6 of which were previously published in Fu et al. [2016], and the remaining 60 samples are as of this writing unpublished (a manuscript for these samples are in preparation, Yu et al.). We compared hapCon

and ANGSD on these samples and found that contamination estimates obtained from the two methods are highly concordant (r^2=0.8347, Fig.S13). Additionally, we investigated the difference between the estimates of our method and that of ANGSD for various sample ages (the mean of the posterior interval of the C14 date or the mean of the archaeological context range). A regression line with slope equal to 0 would indicate that the bias of our method does not systematically change with sample age. Our results indicate that, while nominally the regression line has non-zero negative slope (-1.22e-6), the p_value is not significant (0.059). If we restrict fitting the regression to samples with coverages greater than 0.1x (51 out of 66 samples), the p_value becomes 0.25. Therefore, there is no significant evidence that the performance of our method is more biased for older Eurasian samples, which aligns with our previous results.

Second, we performed mixed-BAM simulation on three of the higher coverage samples in Fu et al. [2016]; namely, GoyetQ116-1, Kostenki14 and Vestonice16. We used the three samples as the endogenous source and B_French-3 from SGDP as the contamination source. We simulated contamination rates from 0% to 25% and various coverages. We found that hapCon's results are comparable to ANGSD at high coverage and low contamination regime, and much better at low coverage and high contamination regime, showing no systematic bias despite the fact that these samples contain highly divergent genetic ancestries (Fig.S15, S16, S17).

4.2 Ancient African Foragers

In the main text we tested hapCon on Mota, an ancient African genome from present-day Ethiopia that predates the Eurasian genetic backflow. We observed a small amount of overestimation (~0.7%) compared with ANGSD. Here we conducted further tests on African forager genomes known to harbor the most divergent lineages of all living peoples. We used ten male ancient African foragers from Lipson et al. [2022], among which I8930, I13983 and I19529 do not have sufficient coverage for estimating contamination by ANGSD. For the remaining seven male samples, we summarized the results in Tab.S2.

For four samples out of the seven samples with sufficient coverages, we obtained consistent contamination estimates for hapCon and ANGSD. For the three samples from present-day Malawi (I4427, I4468, I19528, corresponding rows highlighted in grey), we observe that hapCon

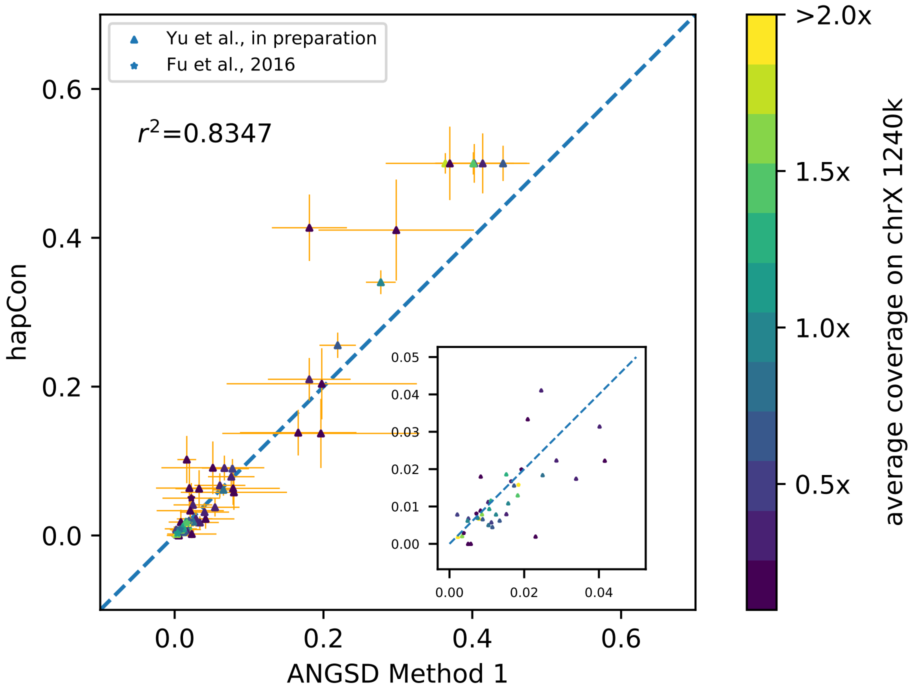

Figure S13: **Comparing hapCon and ANGSD on 66 Eurasian Hunter-gatherers** Same as Fig.5d, but this figure includes the Eurasian hunter-gatherers only.

Table S2: **Comparing ANGSD and hapCon on Ancient African Foragers**

Sample ID	Site	Age(years BP)	Coverage(1240k target on chrX)	ANGSD Method1	hapCon
I10871	Shum Laka	7975-7795	10.33x	0.008(0.007-0.008)	0.006(0.005-0.006)
I10872	Shum Laka	7920-7700	1.85x	0.016(0.014-0.019)	0.016(0.015-0.018)
I10873	Shum Laka	3160-2970	9.14x	0.007(0.006-0.007)	0.004(0.004-0.005)
I8821	Kisese II RS	7240-6985	1.44x	0.006(0.004-0.008)	0.007(0.005-0.008)
I4427	Fingira	6175-5930	0.11x	0.01(-0.007-0.028)	0.052(0.035-0.069)
I4468	Fingira	6180-5935	0.063x	-0.016(-0.034-0.003)	0.064(0.035-0.093)
I19528	Hora 1	16424-14029	0.075x	0.01(-0.009-0.029)	0.062(0.039-0.086)

flags these samples as being moderately contaminated while ANGSD's estimates suggest that they are at most minimally contaminated. Aside from the relatively low coverage of I4468 and I19528, which is at the boundary of ANGSD's working coverage, we believe the inconsistency is at least partially due to some of the deeply diverged ancestry of these central-south African foragers not being represented well in the 1000Genome reference panel. Based on qpAdm analysis, the three samples (I4427, I4468, I19528) from present-day Malawi trace 20-30% of their ancestry to Mota-related, 5-10% to central-African related and 60-70% to southern-African related ances-

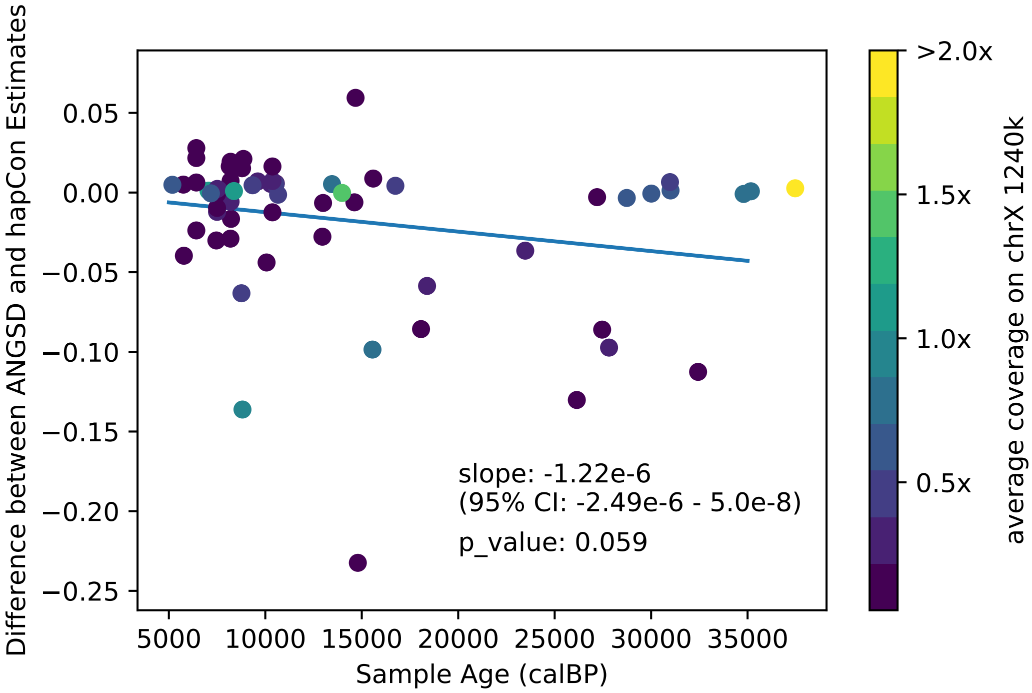

Figure S14: **Comparison of hapCon and ANGSD for varying sample age** We plotted the difference between hapCon and ANGSD contamination estimates for the 66 Eurasian Hunter-gatherer samples (see also Fig.S13), with the x axis denoting the age of each sample. We fit a linear regression to the data. The p_value corresponds to the null hypothesis of the slope being 0.

try [Lipson et al., 2022]. As southern-African ancestry are not represented in 1000Genome reference panel and the three African ancestries (southern Africa, central Africa and northeastern Africa) form three distinct clusters on a PCA plot (Fig 1b, Lipson et al. [2022]), it is not surprising that a haplotype copying model does not work well on these ancient forager genomes. In addition, the three samples for which hapCon's results are not consistent with those of ANGSD are the three with the lowest coverage of the seven samples. A potential explanation is that hapCon is more reliant on the haplotpye copying model in the low coverage regime. In higher coverage regimes there are more sites covered by more than one sequences and the information from the majority sequences can compensate for the endogenous haplotpye not being well-modeled as a haplotype mosaic of the reference panel.

To provide further support (aside from Mota) that our method can work for African aDNA

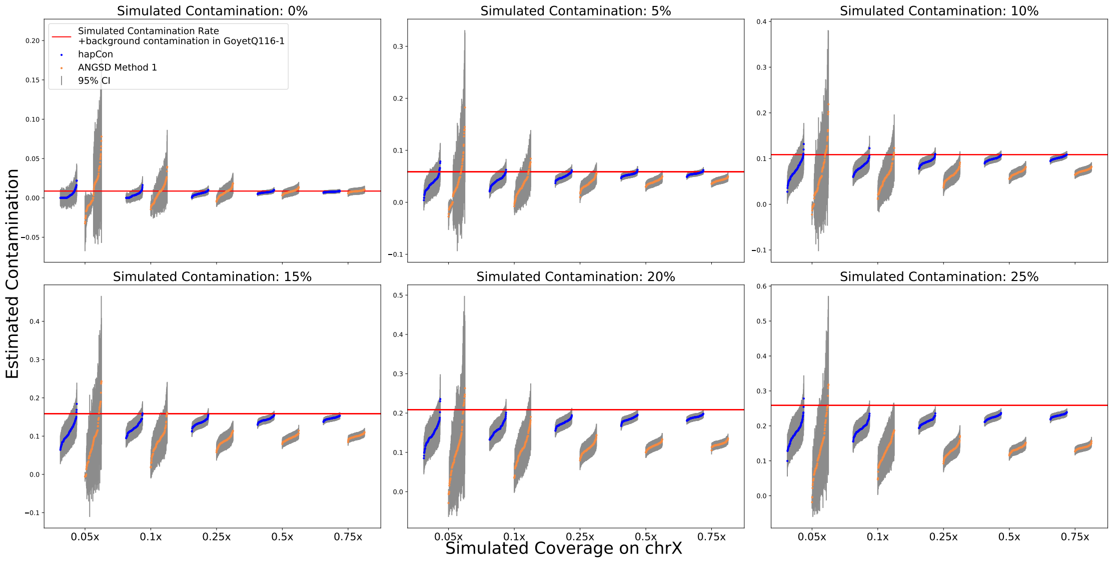

Figure S15: **Simulating Contamination Using GoyetQ116-1 as the Endogenous Source**

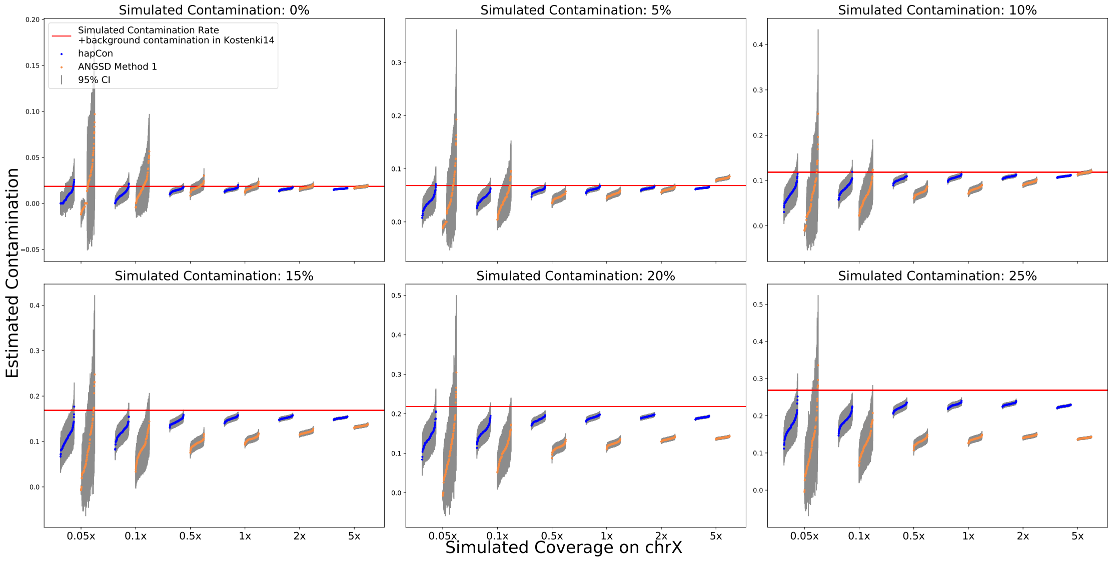

Figure S16: **Simulating Contamination Using Kostenki14 as the Endogenous Source**

data (except for the southern forager ancestry highlighted above), we additionally performed mixing BAM simulation using the sample with the highest coverage from Lipson et al. [2022]. We used I10871 as the endogenous source and B_French-3 from SGDP as the contamination source, and we visualized the results in Fig.S18. We observe that hapCon's performance on

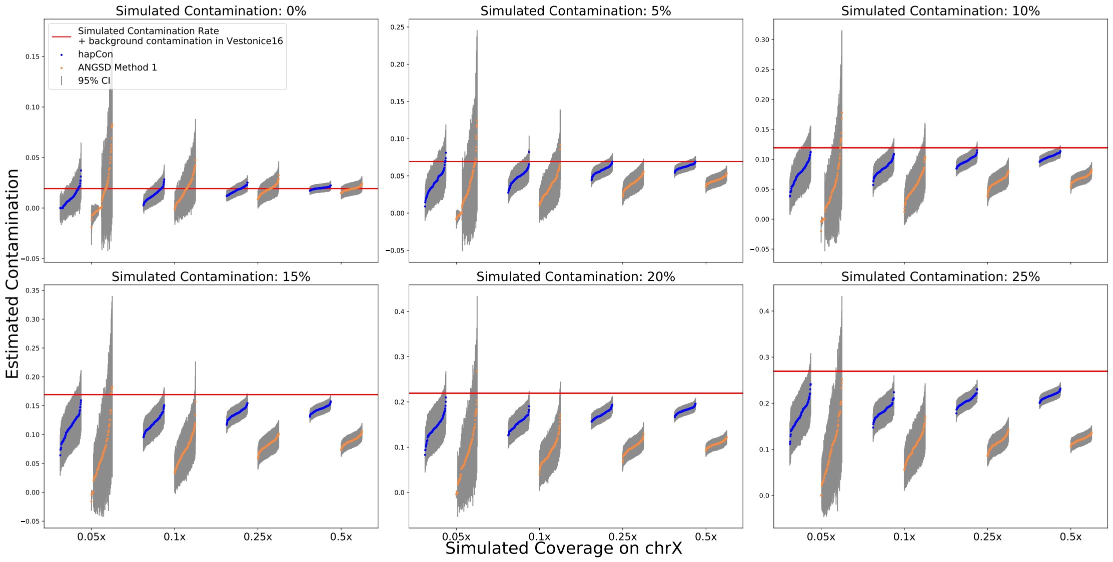

Figure S17: **Simulating Contamination Using Vestonice16 as the Endogenous Source**

I10871 is as good as for Eurasian samples and generally outperforms ANGSD.

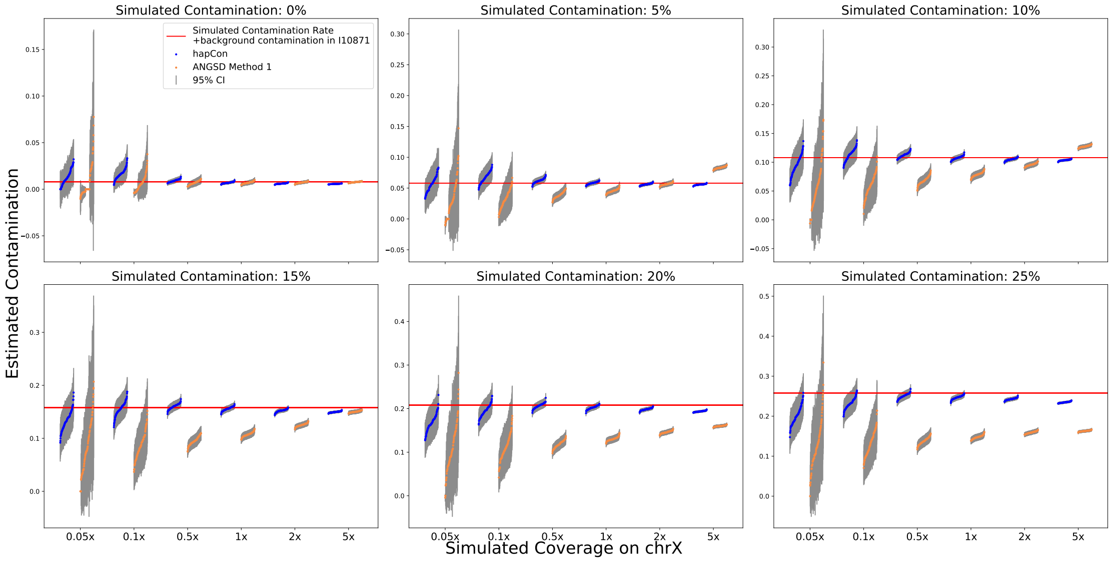

Figure S18: **Simulating Contamination Using I10871 as the Endogenous Source**

Overall, these analyses suggest that caution is warranted for interpreting hapCon contamination estimates if the sample derive substantial ancestry from deeply divergent lineages (prior to the out-of-Africa event) that are not represented in the 1000G reference dataset. In that case,

we advise caution and suggest to use ANGSD or the two-consensus method (if coverage suffices).

5 Performance on Medium-to-high Coverage Data

We observe that, for the same contamination level and sample, contamination estimates with hapCon tend to increase for higher coverage. Here we explore whether this phenomenon lead to substantial biases in the high coverage regimes and whether it would affect the qualitative assessment of a sample being highly contaminated or not.

We used Ust_Ishim as the endogenous source and B_French-3 from SGDP as the contaminant sources. We simulated contamination rate ranging from 0% to 25% in steps of 5% at coverages 1x, 2x, 5x, 10x, 15x. As throughout, coverages always refer to average coverage on male chrX. We note that the full data of UstIshim on chrX is 18x (after quality filtering), thus the replicates are not fully independent anymore in the high coverage regime.

We applied hapCon with both the 1240k and 1000G panel on these simulated data, and visualized the results in Fig.S19,S20,S21. We observe that both hapCon (Fig.S19,S20) and ANGSD (Fig.S21) display some degree of varying biases associated with coverage. In most cases the biases decrease as the coverages increases, but also generally do not seem to converge to 0, possibly due to deviations from the model assumptions (e.g. read counts being modeled by the binomial distribution, or contamination coming from an average and perfectly specified allele frequency). However, the biases remain small for both methods and would not affect the qualitative assessment of whether a sample is contaminated or not.

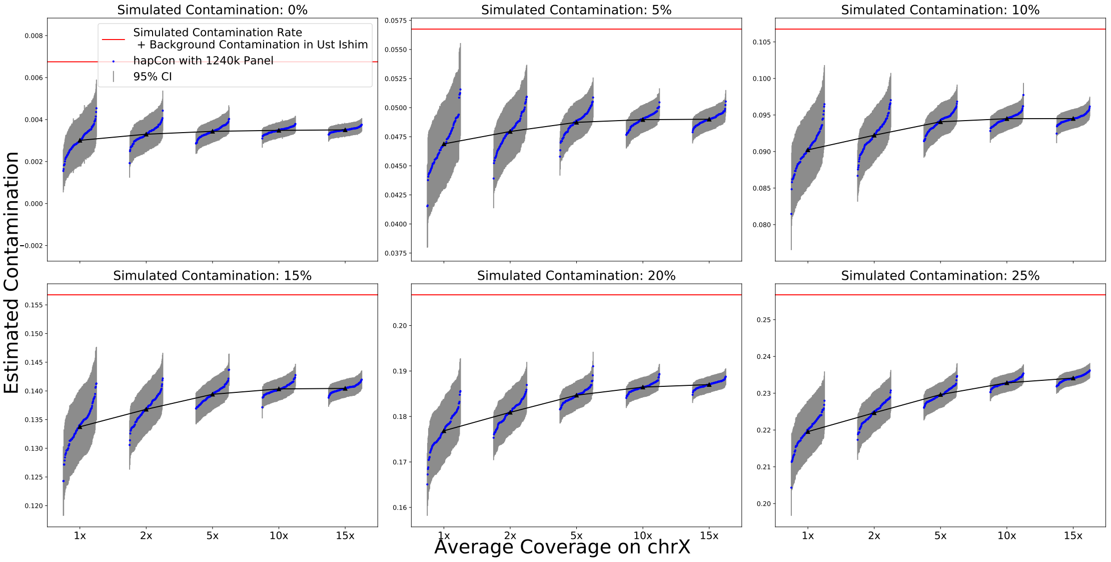

Figure S19: **Performance of hapCon with 1240k Panel at High Coverage.** Note the changing Y axis scale (adapted to the range of estimates). The red horizontal line represents the simulated contamination rate plus the ANGSD estimate of background contamination in the Ust_Ishim BAM file (0.675%, 95% CI: 0.637%-0.713%). We also visualized trend of estimated contamination as a function of coverage by connecting the dots that represent the mean value of estimated contamination (averaged across 100 replicates).

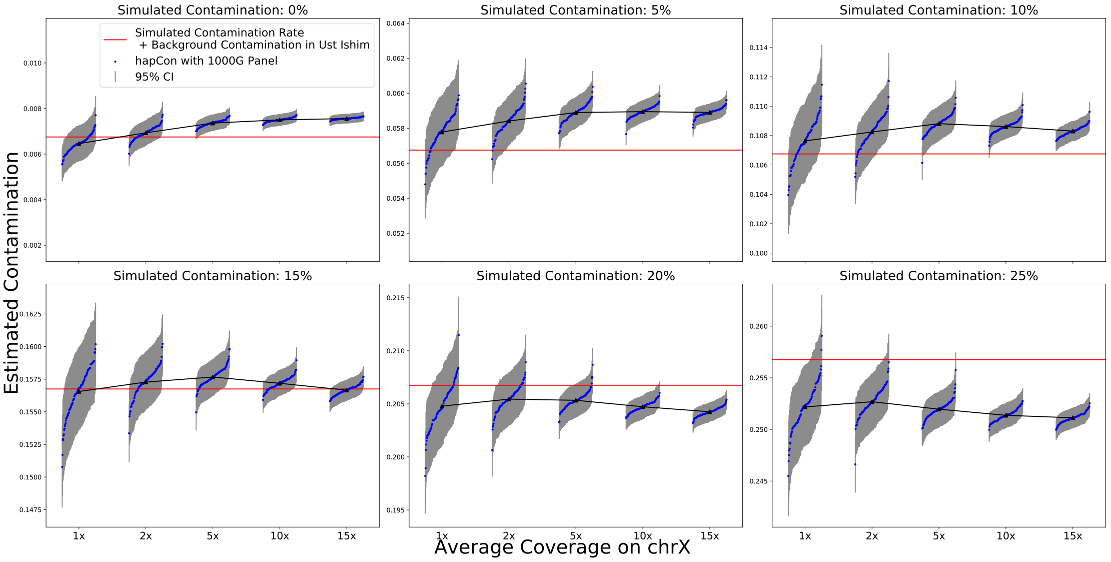

Figure S20: **Performance of hapCon with 1000G Panel at High Coverage.** Note the changing Y scale (adapted to the estimates).

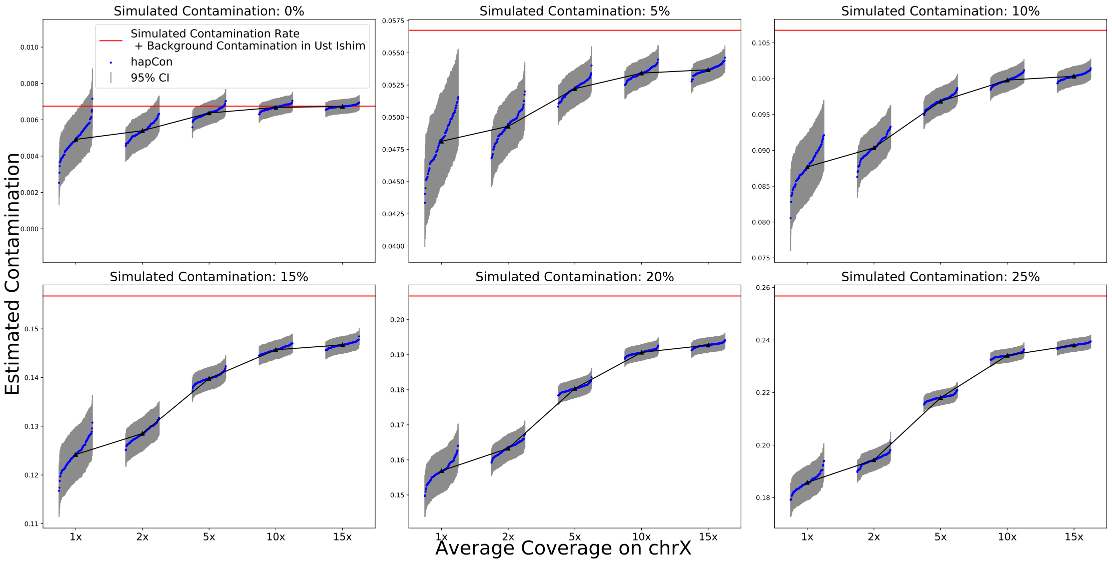

Figure S21: **Performance of ANGSD at High Coverage.** Note the changing Y scale (adapted to the estimates).

6 Supplementary Figures

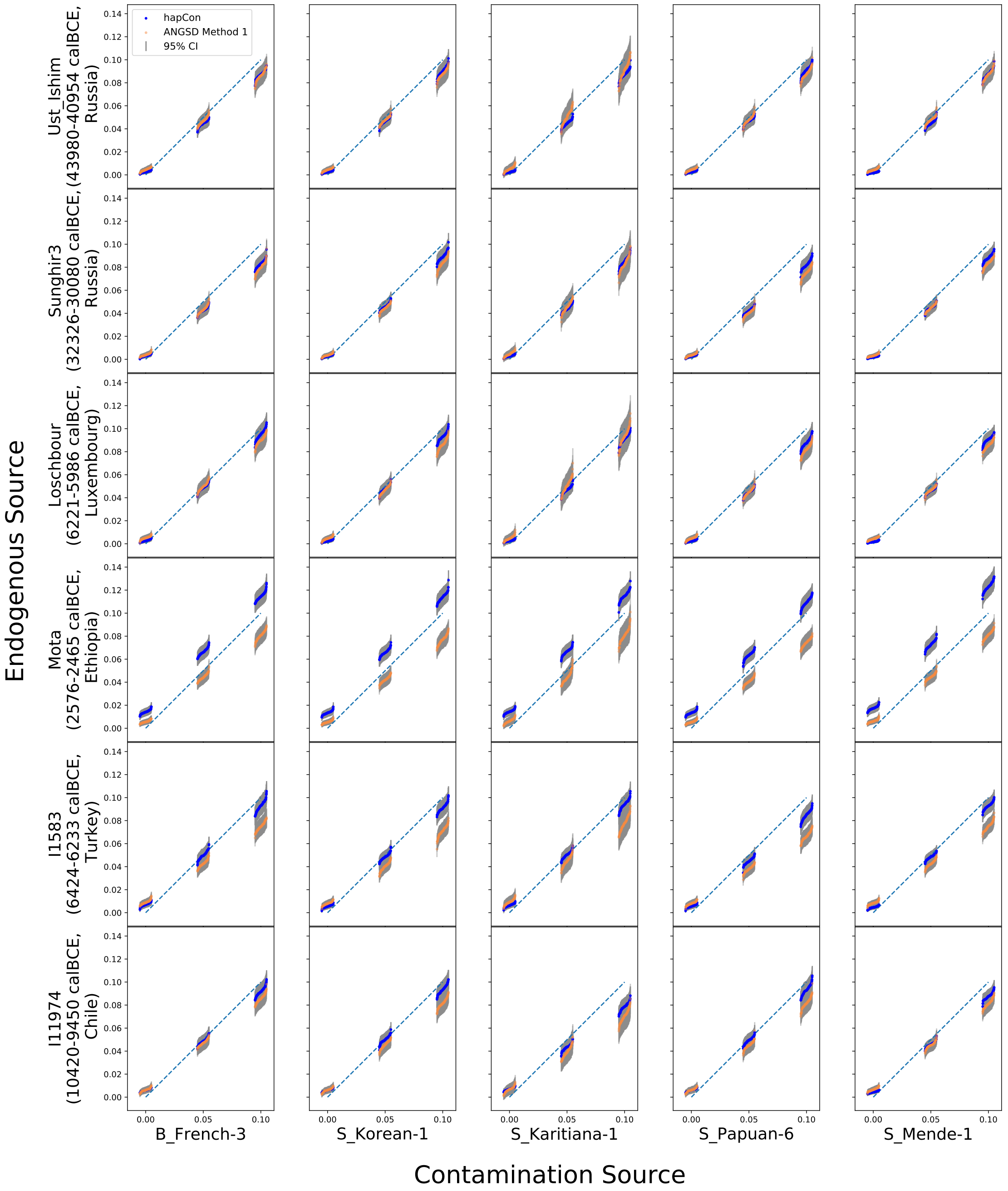

Figure S22: **Zoom-in for Fig.2 in main text** This is a zoom-in into simulated contamination in the range of 0%-10% for the Fig.2 in the main text.

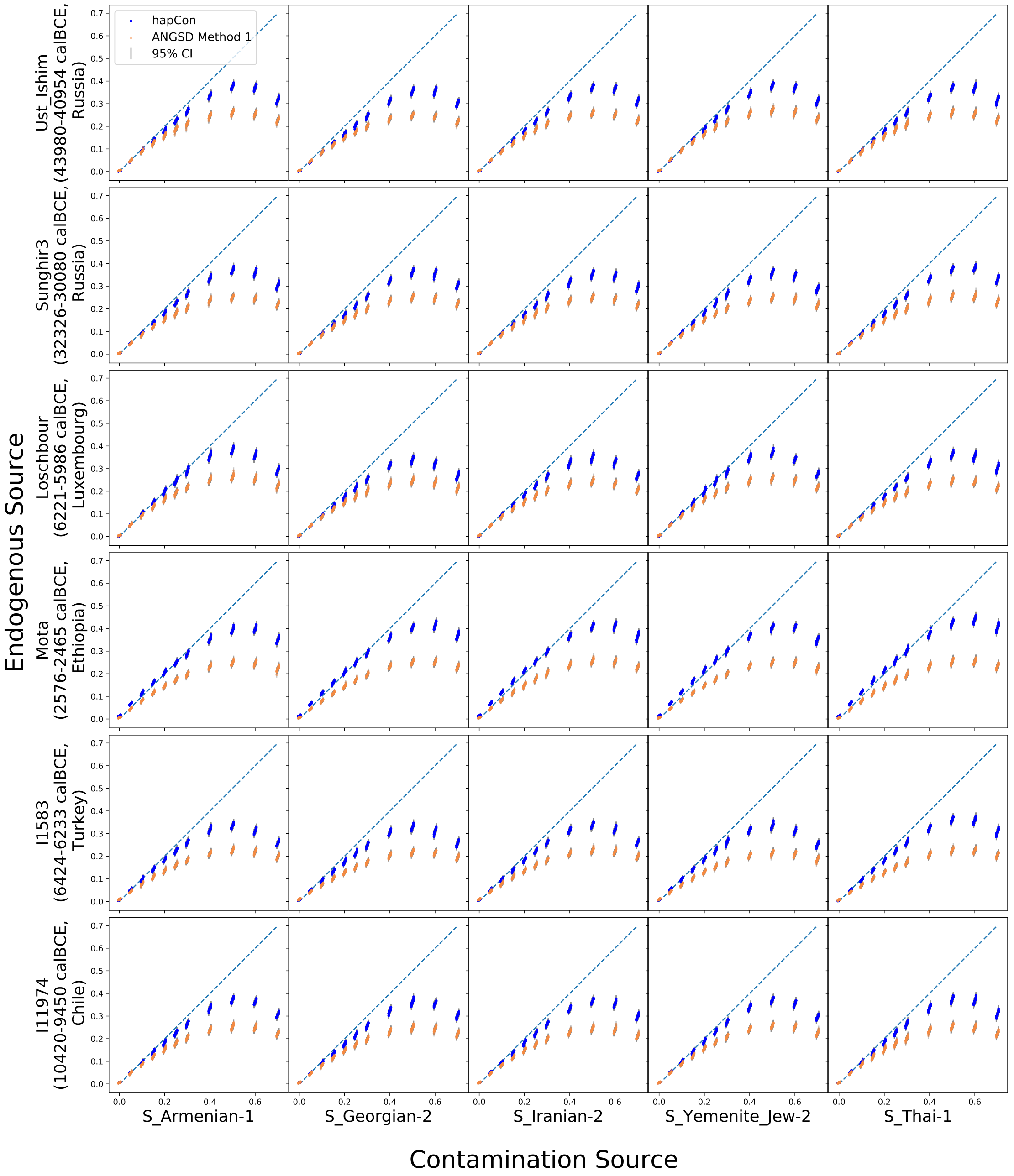

Figure S23: **Testing hapCon on Various Contamination and Endogenous Sources** This figure has the same structure as Fig.2 in the main text, but with 5 different contamination sources that are less well-represented in the 1000Genome Project.

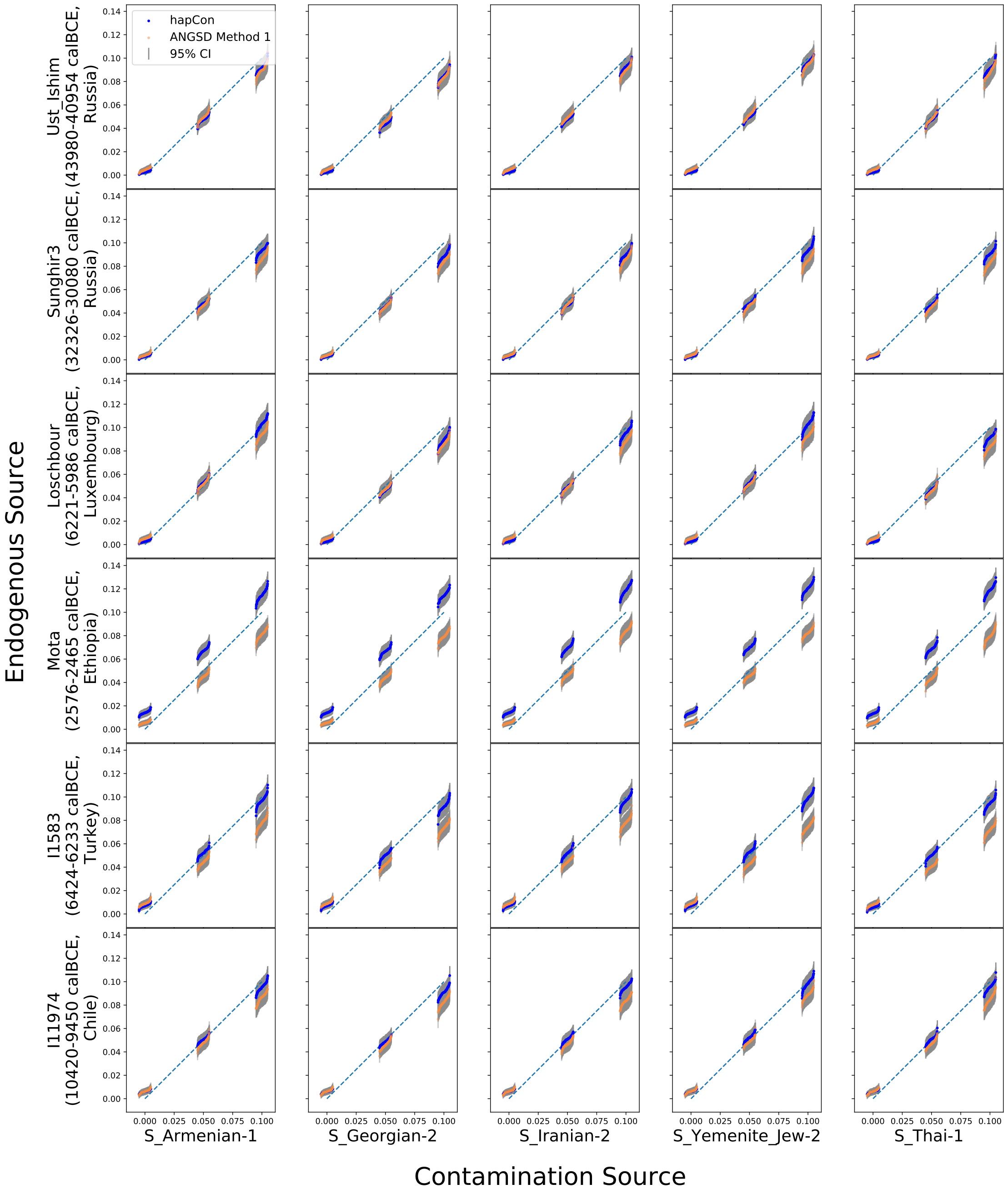

Figure S24: **Zoom-in for Fig.S23**

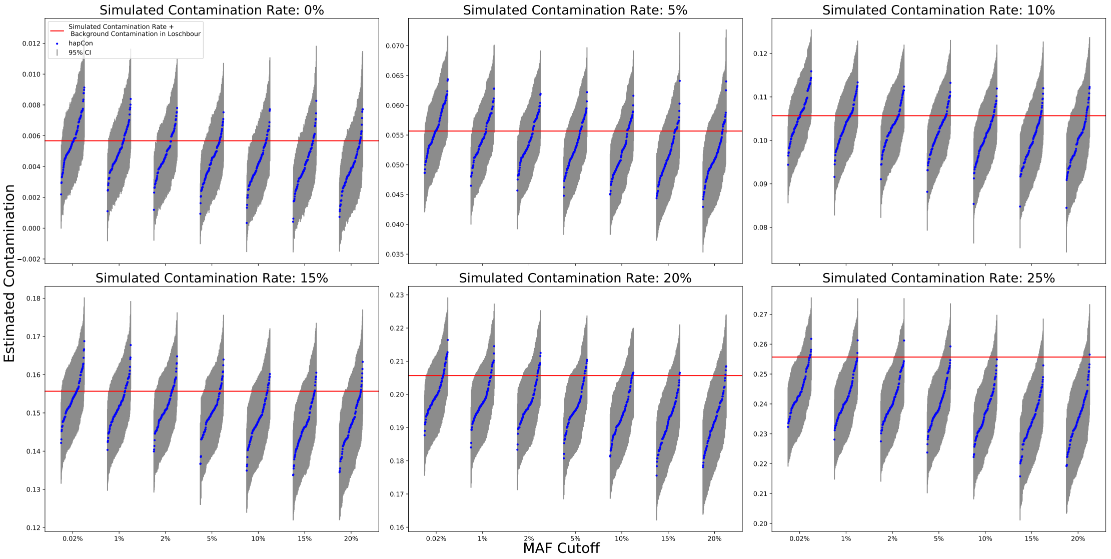

Figure S25: **Comparing hapCon on 1000G Panel with Different MAF Cutoff at Various Contamination Level.** We performed mixed BAM simulation (use Loschbour as the endogenous source and B_French-3 as the contaminant source) at coverage 0.1x as described in the main article. We compared hapCon's performance on the 1000G panel with varying minor allele frequency cutoffs.

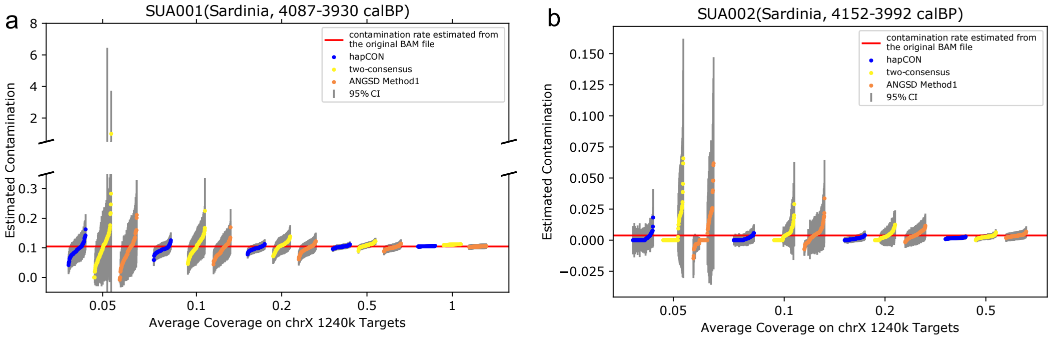

Figure S26: **Comparing hapCon, the two-consensus method and ANGSD on Downsampled Sardinia aDNA data.** Downsampling was performed as described in the main article. We compare the performance of our method, the two-consensus method and ANGSD. **a** Comparison on individual SUA001. **b** Comparison on individual SUA002.

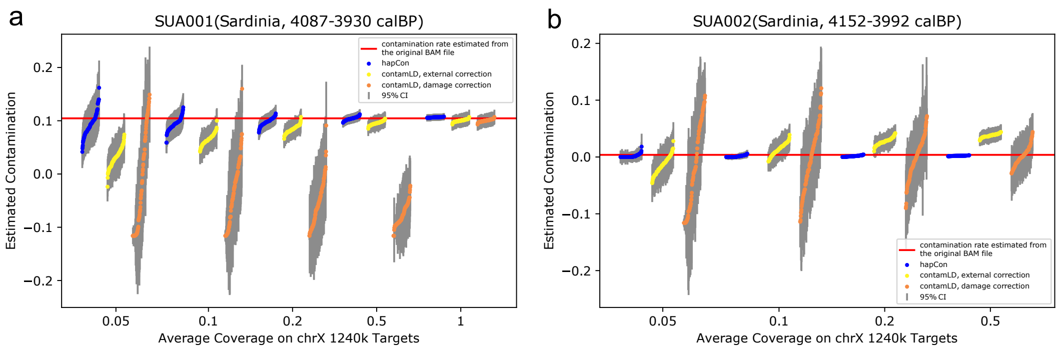

Figure S27: **Comparing hapCon and contamLD on Downsampled Sardinia aDNA data.** Downsampling was performed as described in the main article. We compare the performance of hapCon and contamLD. CEU is used as the reference panel for contamLD. **a** Comparison on individual SUA001. **b** Comparison on individual SUA002.

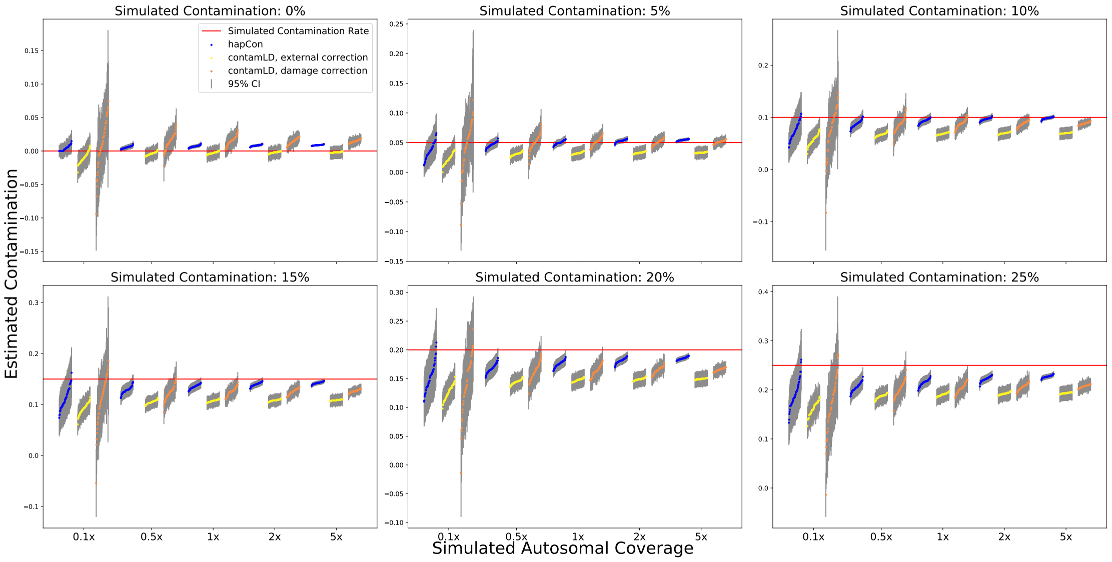

Figure S28: **Comparing contamLD and hapCon on simulated Data.** We simulated different levels of contamination by mixing BAM files of I1583(6424-6233 calBEC, Turkey) and B_French_3 and then downsampling to desired genome-wide coverage. Note that the x-axis refers to average coverage on autosomes, unlike the other figures in this manuscript where the coverage always refers to coverage on male X chromosome. For each simulated scenario, we made 50 independent replicates and then applied contamLD (using CEU as the reference panel) and hapCon to the data.

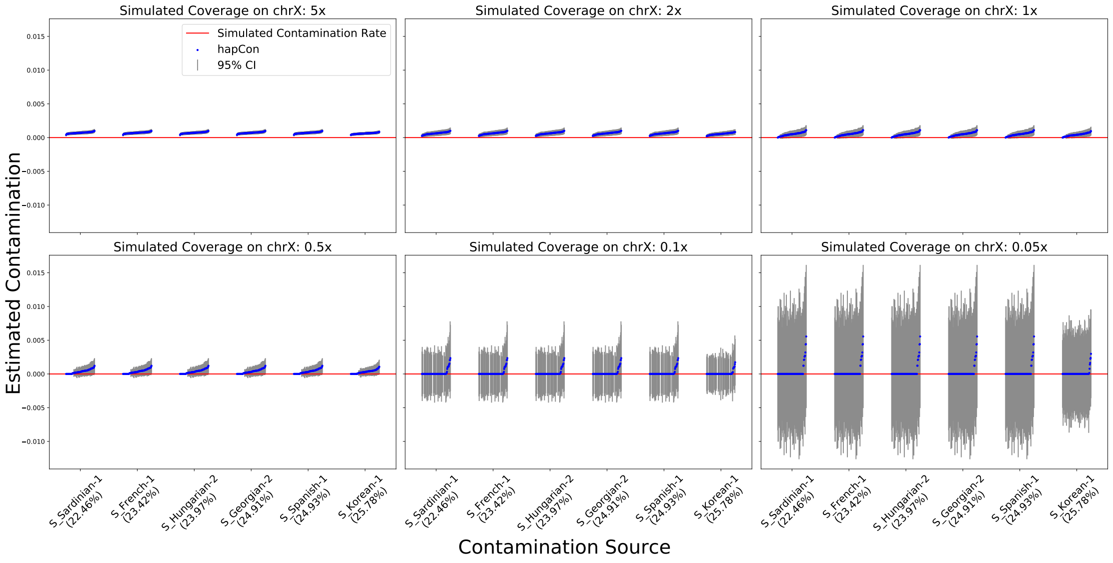

Figure S29: **Effects of Genetic Similarity between the Endogenous and Contaminant Source on Contamination Estimation for Simulated 0% Contamination** We used B_French-3 as the endogenous source and used as contamination source S_Sardinian-1, S_French-1, S_Hungarian-2, S_Georgian-2, S_Spanish-1, S_Korean-1, which are indicated on the x-axis. The percentage number in the parenthesis of x-labels are genetic distances between the endogenous and contaminant sources, calculated as described in the main article. We mixed the BAM files of the endogenous and contaminant source and downsampled to desired genome-wide coverage. We then analyzed the mixed BAM files using hapCon with 1240k panel. For S_Korean-1 we used CHB allele frequency as a proxy, and for all the others we used CEU allele frequency. This figure visualized the results for simulated 0% contamination.

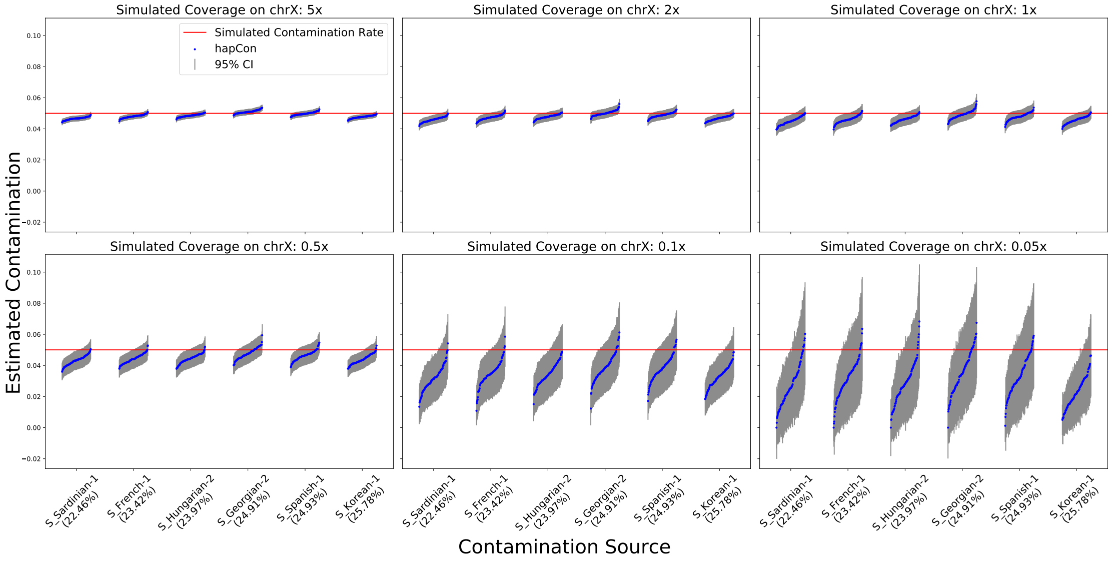

Figure S30: **Effects of Genetic Similarity between the Endogenous and Contaminant Source for Simulated 5% Contamination** Same as Fig.S29, but with 5% simulated contamination.

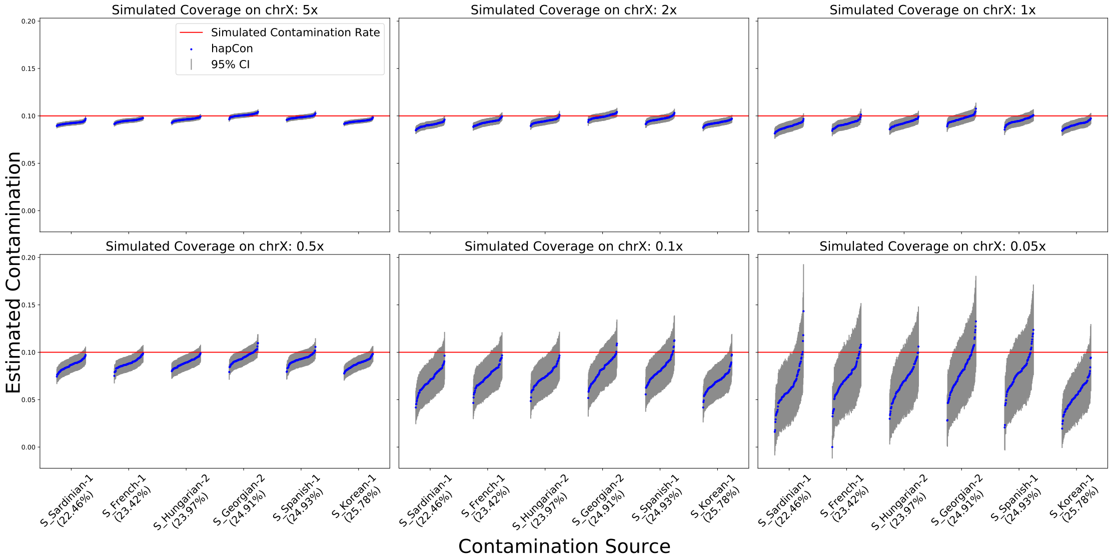

Figure S31: **Effects of Genetic Similarity between the Endogenous and Contaminant Source for Simulated 10% Contamination** Same as Fig.S29, but with 10% simulated contamination.

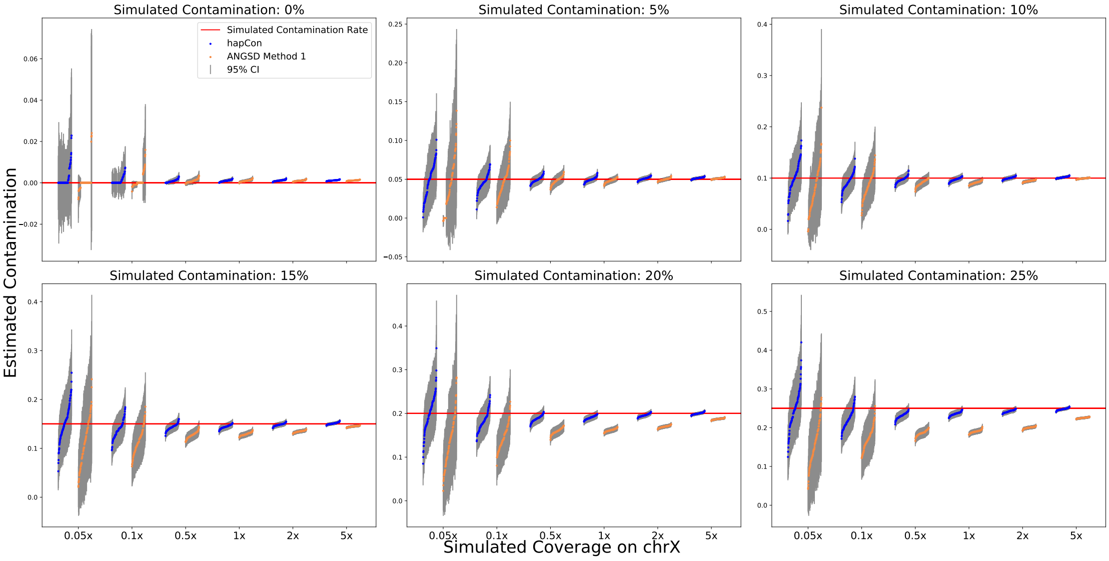

Figure S32: **Japanese contaminated with Japanese at vaiours levels of Contamination and Coverages** S_Japanese-1 is contaminated with S_Japaneses-3, both samples are from SGDP. We simulated contamination levels from 0% to 25% at various coverages from 0.05x to 5x, and compared results from hapCon and ANGSD.

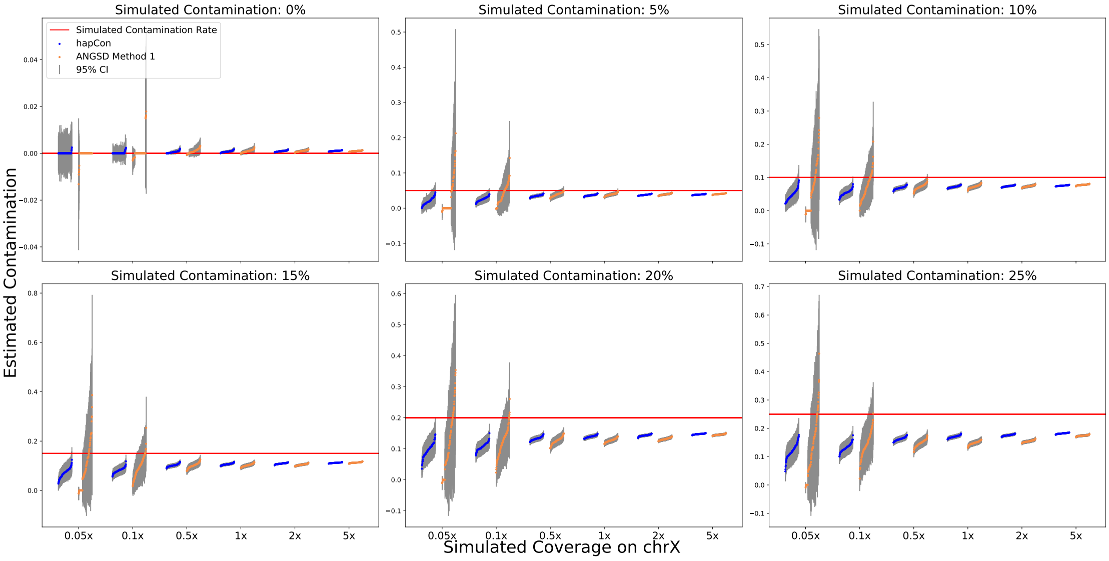

Figure S33: **Karitiana contaminated with Karitiana at various levels of Contamination and Coverages** B_Karitiana-3 is contaminated with S_Karitiana-1, both samples are from SGDP. We simulated contamination levels from 0% to 25% at various coverages from 0.05x to 5x, and compared results from hapCon and ANGSD.

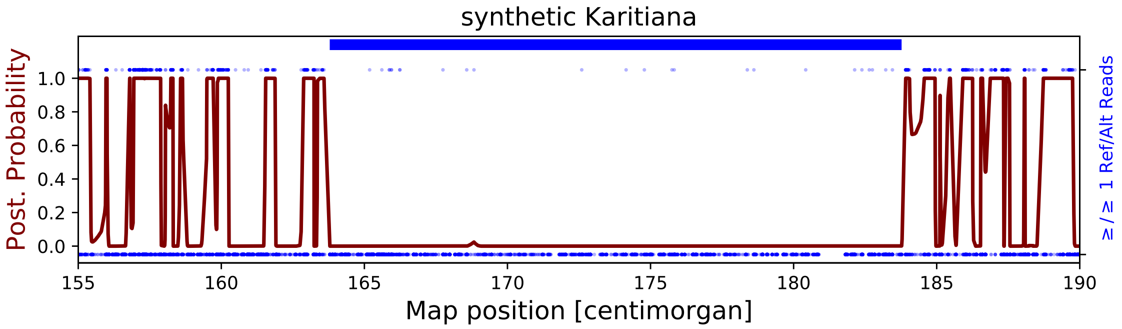

Figure S34: **Visualizing long IBD on chrX between Two Karitiana Samples** We downsampled the BAM files of B_Karitiana-3 and S_Karitiana-1 both to 15x and merged them together. As the two Karitiana samples are both males, this results in a synthetic diploid X chromosome. We then applied hapROH [Ringbauer et al., 2021] to detect ROH blocks in this synthetic diploid X chromosome, which is equivalent to IBD between the two haploid X chromosome of the two male Karitiana samples. We found a 19.98cM(163.79cM-183.77cM) long IBD block and visualized it here. The brown curve depicts the posterior probability of being in non-IBD state at each of the 1240 target SNPs, and the blue dots at the bottom depicts the marker density along the chromosome. The blue dots at the top represent potentially heterozygote sites. Each site that have at least one sequence supporting both the reference and alternative alleles is represented by a blue dot at the top. Due to several sources of errors (e.g, sequencing error, mismappings), there are several apparent "heterozygous" sites in the IBD region; however, such "heterozygous" sites in IBD region are much more sparse than that in the non-IBD region. The horizontal blue bar at the very top of the figure is the inferred IBD block.

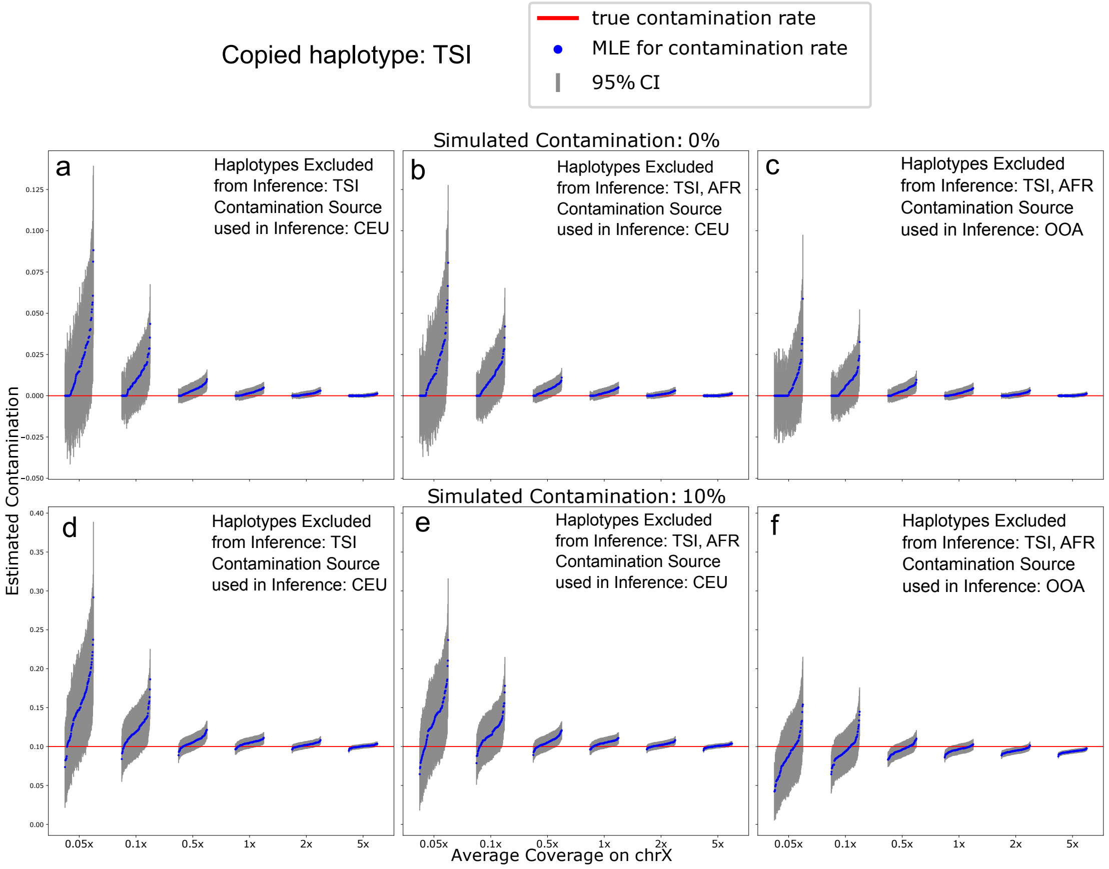

Figure S35: **Attraction to Contaminant Allele Frequency when the Contaminant and Global Allele Frequencies in the Reference Panel are different.** We performed the simulation using the default setting as described in Section 2.1 except that we used the CEU as the contamination source (rather than the global allele frequencies). Panels **a-c** show the results for no simulated contamination and panels **d-f** for simulated 10% contamination. We explored several different settings for inference by removing divergent haplotypes (AFR) from the reference panel and by using different allele frequencies as the proxy of the contamination source (CEU vs. OOA, where OOA denotes the allele frequencies of all populations in the 1000Genome except for AFR). Settings are indicated in the upper right corner of each subpanel.

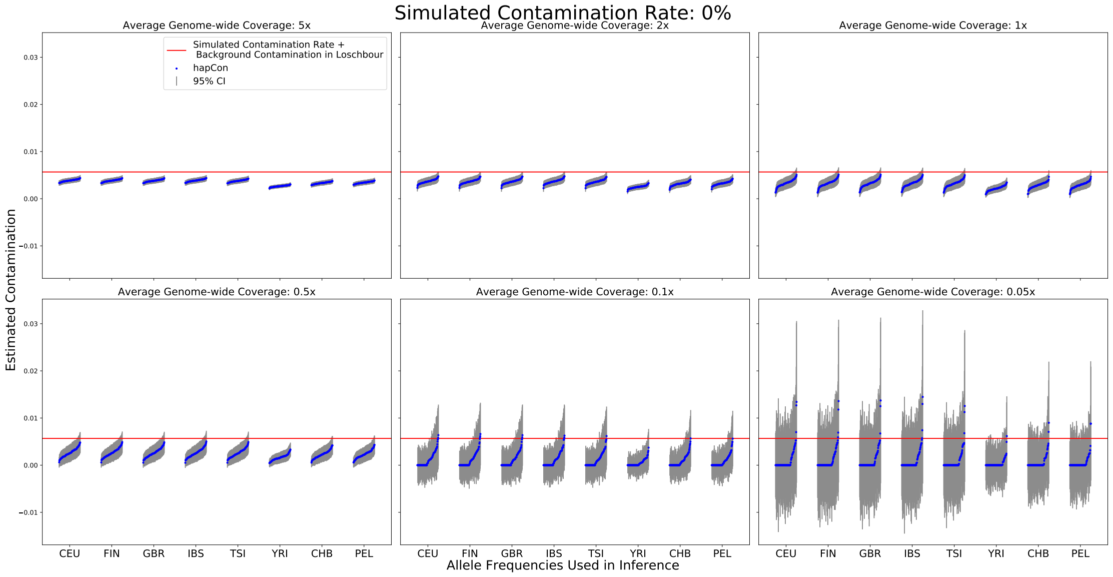

Figure S36: **Misspecified Contamination Ancestry in Mixed BAM Simulation with 0% Simulated Contamination.** We used the same mixed BAM simulation as described in section ”Simulated whole genome sequencing data” in the main article (using Loshcbour as the endogenous source and B_French-3 as the contaminant source). For each coverage, we used the 1240k reference panel with CEU, FIN, GBR, IBS, TSI, YRI, CHB, PEL as the contamination ancestry to test the robustness of our method with respect to mis-specified contamination ancestry.

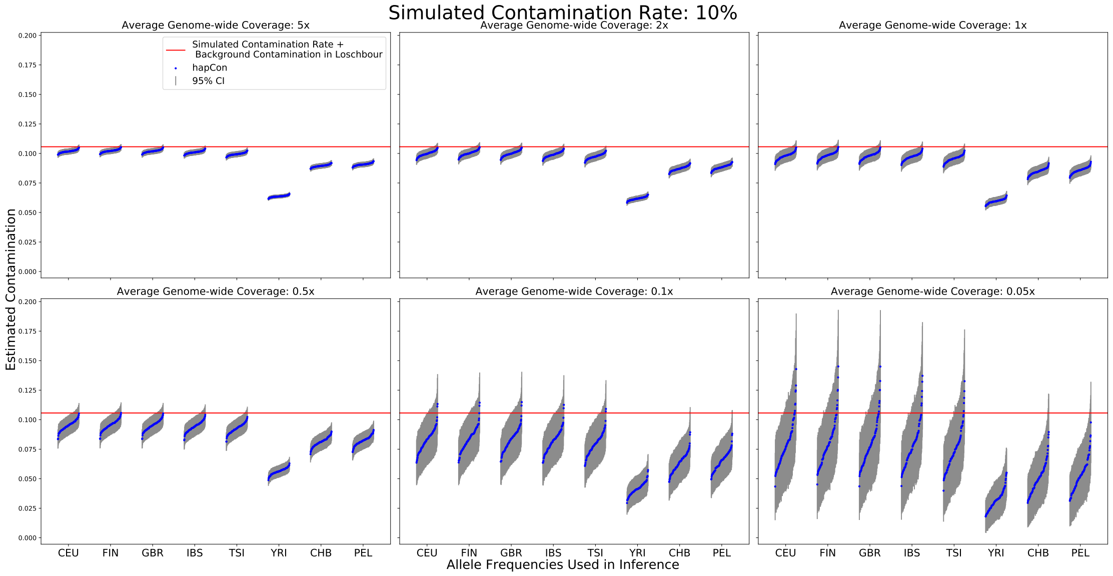

Figure S37: **Misspecified Contamination Ancestry in Mixed BAM Simulation with 10% Simulated Contamination.** Same as Fig.S36, but with 10% simulated contamination.

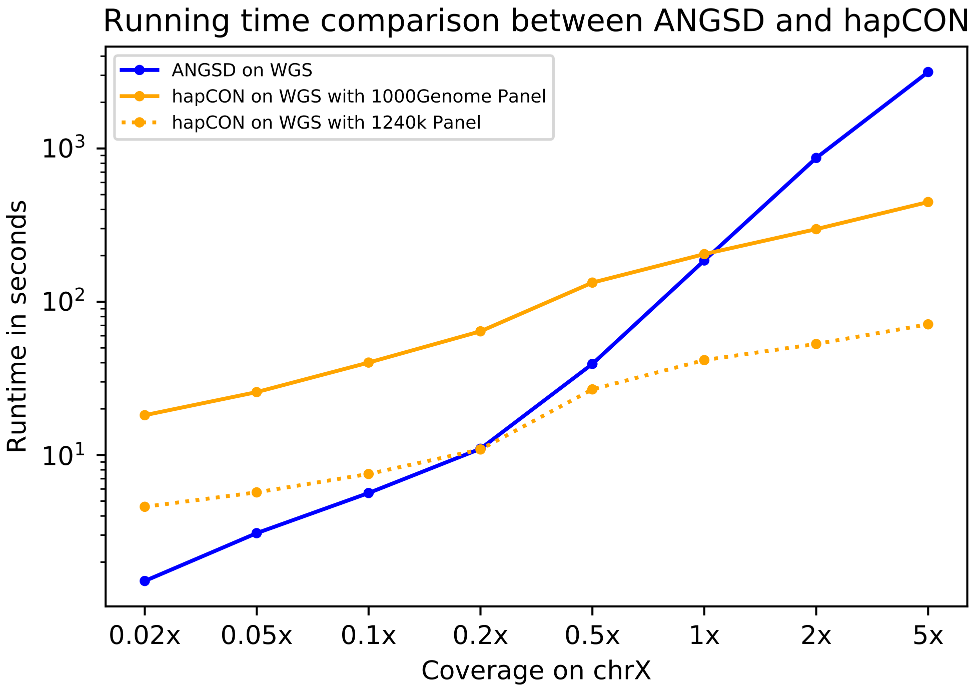

Figure S38: **Comparing runtime of hapCon and ANGSD.** We measured runtime of hapCon and ANGSD on BAM files of individual I1496(5211-4958 calBCE, Hungary, obtained from Allen Genome Diversity Project), down-sampled to eight target coverages. For hapCon, we used two different reference panels (1240k and 1000G panel). Each point represents the runtime averaged over 10 independent runs.

References

Arjun Biddanda, Matthias Steinrücken, and John Novembre. Properties of two-locus genealogies and linkage disequilibrium in temporally structured samples. *bioRxiv*, 2021.

1000 Genomes Project Consortium et al. A global reference for human genetic variation. *Nature*, 526(7571):68, 2015.

Qiaomei Fu, Cosimo Posth, Mateja Hajdinjak, Martin Petr, Swapan Mallick, Daniel Fernandes, Anja Furtwängler, Wolfgang Haak, Matthias Meyer, Alissa Mittnik, et al. The genetic history of ice age europe. *Nature*, 534(7606):200–205, 2016.

Mateja Hajdinjak, Fabrizio Mafessoni, Laurits Skov, Benjamin Vernot, Alexander Hübner, Qiaomei Fu, Elena Essel, Sarah Nagel, Birgit Nickel, Julia Richter, et al. Initial upper palaeolithic humans in europe had recent neanderthal ancestry. *Nature*, 592(7853):253–257, 2021.

Hákon Jónsson, Aurélien Ginolhac, Mikkel Schubert, Philip LF Johnson, and Ludovic Orlando. mapdamage2. 0: fast approximate Bayesian estimates of ancient DNA damage parameters. *Bioinformatics*, 29(13):1682–1684, 2013.

Iosif Lazaridis, Nick Patterson, Alissa Mittnik, Gabriel Renaud, Swapan Mallick, Karola Kirsanow, Peter H Sudmant, Joshua G Schraiber, Sergi Castellano, Mark Lipson, et al. Ancient human genomes suggest three ancestral populations for present-day europeans. *Nature*, 513(7518):409–413, 2014.

Mark Lipson, Elizabeth A Sawchuk, Jessica C Thompson, Jonas Oppenheimer, Christian A Tryon, Kathryn L Ranhorn, Kathryn M de Luna, Kendra A Sirak, Iñigo Olalde, Stanley H Ambrose, et al. Ancient dna and deep population structure in sub-saharan african foragers. *Nature*, pages 1–7, 2022.

Po-Ru Loh, Petr Danecek, Pier Francesco Palamara, Christian Fuchsberger, Yakir A Reshef, Hilary K Finucane, Sebastian Schoenherr, Lukas Forer, Shane McCarthy, Goncalo R Abecasis, et al. Reference-based phasing using the haplotype reference consortium panel. *Nature Genetics*, 48(11):1443–1448, 2016.

Swapan Mallick, Heng Li, Mark Lipson, Iain Mathieson, Melissa Gymrek, Fernando Racimo, Mengyao Zhao, Niru Chennagiri, Susanne Nordenfelt, Arti Tandon, et al. The Simons genome diversity project: 300 genomes from 142 diverse populations. *Nature*, 538(7624): 201–206, 2016.

J Víctor Moreno-Mayar, Thorfinn Sand Korneliussen, Jyoti Dalal, Gabriel Renaud, Anders Albrechtsen, Rasmus Nielsen, and Anna-Sapfo Malaspinas. A likelihood method for estimating present-day human contamination in ancient male samples using low-depth X-chromosome data. *Bioinformatics*, 36(3):828–841, 2020.

Iñigo Olalde, Swapan Mallick, Nick Patterson, Nadin Rohland, Vanessa Villalba-Mouco, Marina Silva, Katharina Dulias, Ceiridwen J Edwards, Francesca Gandini, Maria Pala, et al. The genomic history of the Iberian Peninsula over the past 8000 years. *Science*, 363 (6432):1230–1234, 2019.

Kay Prüfer, Cosimo Posth, He Yu, Alexander Stoessel, Maria A Spyrou, Thibaut Deviese, Marco Mattonai, Erika Ribechini, Thomas Higham, Petr Velemínskỳ, et al. A genome sequence from a modern human skull over 45,000 years old from Zlatỳ kůň in Czechia. *Nature ecology & evolution*, 5(6):820–825, 2021.

Morten Rasmussen, Xiaosen Guo, Yong Wang, Kirk E Lohmueller, Simon Rasmussen, Anders Albrechtsen, Line Skotte, Stinus Lindgreen, Mait Metspalu, Thibaut Jombart, et al. An Aboriginal Australian genome reveals separate human dispersals into asia. *Science*, 334 (6052):94–98, 2011.

Gabriel Renaud, Kristian Hanghøj, Eske Willerslev, and Ludovic Orlando. gargammel: a sequence simulator for ancient DNA. *Bioinformatics*, 33(4):577–579, 2017.

Harald Ringbauer, John Novembre, and Matthias Steinrücken. Parental relatedness through time revealed by runs of homozygosity in ancient DNA. *Nature Communications*, 12(1):1–11, 2021.

Richard J Rossi. *Mathematical statistics: an introduction to likelihood based inference*, page 267. John Wiley & Sons, 2018.

Simone Rubinacci, Diogo M Ribeiro, Robin J Hofmeister, and Olivier Delaneau. Efficient phasing and imputation of low-coverage sequencing data using large reference panels. *Nature Genetics*, 53(1):120–126, 2021.
